# Phytochemical Investigation of *Crataegus almaatensis* Leaves: Isolation, Structural Characterization, and Antioxidant Activity of Flavonoids, Flavolignans, and Phenolic Glycosides

**DOI:** 10.3390/antiox15091149

**Published:** 2026-09-10

**Authors:** Zhanar Nabiyeva, Akerke Kulaipbekova, Asylbek Kozybayev, Handi Yang, Fuhang Song, Abdyssemat Samadun, Elmira Assembayeva, Nasi Ai

**Affiliations:** 1 Research Institute of Food Safety, Almaty Technological University, Almaty 050012, Kazakhstan; atu_nabiyeva@mail.ru (Z.N.); abdu.93_93@mail.ru (A.S.); 2 Department of Applied Biotechnology, Almaty Technological University, Almaty 050012, Kazakhstan; a.kulaipbekova@atu.edu.kz; 3 Faculty of Biotechnology and Chemical Technologies, Almaty Technological University, Almaty 050012, Kazakhstan; asilbek_k@mail.ru; 4 Key Laboratory of Geriatric Nutrition and Health, Ministry of Education of China, School of Light Industry Science and Engineering, Beijing Technology and Business University, Beijing 100048, China; yanghandi@st.btbu.edu.cn; 5 Key Laboratory of Geriatric Nutrition and Health, Ministry of Education, Beijing Technology and Business University, Beijing 100048, China; ainasi@btbu.edu.cn

**Keywords:** *Crataegus almaatensis*, phytochemistry, flavanols, flavolignans, NMR spectroscopy, LC-MS

## Abstract

A systematic phytochemical investigation of the leaves of *Crataegus almaatensis* Pojark., an endemic hawthorn species native to the mountainous regions of Kazakhstan, was carried out. Multi-step chromatographic fractionation, comprising ultrasound-assisted methanol extraction, sequential liquid–liquid partitioning, silica gel column chromatography, and preparative HPLC, afforded five phenolic compounds as individual, spectroscopically homogeneous constituents from the ethyl acetate fraction. The structures of all isolated compounds were elucidated by comprehensive analysis of high-resolution mass spectrometry (HR-ESI-QTOF-MS) data and one- and two-dimensional NMR spectroscopy (^1^H, ^13^C, HSQC, HMBC) in DMSO-d_6_. The following compounds were identified: (−)-epicatechin, cinchonain Ia, a methoxy- and hydroxy-substituted diaryl ether β-D-glucopyranoside, kakispyrol, and a dimethoxy-substituted diaryl ether β-D-glucopyranoside. The isolated compounds belong to three chemical classes: flavanols, flavolignans, and diaryl ether-type phenolic glycosides. Evaluation of ABTS and DPPH radical-scavenging activity revealed compound-specific antioxidant profiles, with compound **5** (cinchonain Ia) showing activity comparable to the ascorbic acid standard. This study provides, for the first time, a detailed structural characterization of individual phenolic metabolites from *C. almaatensis* leaves and makes a significant contribution to understanding the chemical diversity of this endemic species.

## 1. Introduction

*Crataegus* L. (hawthorn), a member of the *Rosaceae* family, represents one of the most pharmacologically significant plant genera, owing to the richness of its secondary metabolite profile and the wide spectrum of documented biological activities [1,2,3]. The cardioprotective, antioxidant, and anti-inflammatory properties of hawthorn preparations are predominantly attributed to polyphenolic compounds—flavonoids (vitexin, hyperoside), proanthocyanidins, and phenolic acids—generating sustained scientific and applied interest in the development of functional foods and nutraceuticals [1,2]. A comprehensive review by Cui et al. [3] identified over 300 chemical constituents in various *Crataegus* species, including flavonoids, phenolic acids, triterpenes, lignans, and proanthocyanidins, emphasizing that polyphenolic compounds are the primary determinants of the antioxidant, anti-inflammatory, and cardioprotective properties of this genus.

Flavonoids are among the most extensively studied compound groups in *Crataegus*. Edwards et al. [4] provided a detailed account of flavonoid composition, including quercetin derivatives (hyperoside, rutin), vitexin, and its glycosides, all of which exhibit pronounced antioxidant activity as evidenced by their ability to scavenge free radicals and inhibit lipid peroxidation. Comparative LC-MS/MS profiling across different *Crataegus* species and harvesting periods has further confirmed that leaves accumulate consistently high flavonoid levels relative to other plant organs [5], and comprehensive LC-ESI-MS/MS fingerprinting of *C. monogyna* extracts has similarly demonstrated a rich and quantifiable phenolic profile with strong antioxidant activity [6]. Alirezalu et al. [7] demonstrated that *Crataegus* extracts are characterized by high phenolic content and substantial antioxidant activity correlating with flavonoid and procyanidin concentrations, and that oligomeric proanthocyanidins play an important role in vascular protection and improvement of endothelial function. Notably, this antioxidant capacity is not confined to optimal growth conditions: Kirakosyan et al. [8] reported that hawthorn leaf extracts retain, and can even enhance, their polyphenolic antioxidant activity when plants are subjected to environmental stressors such as drought and cold.

Phytochemical research has traditionally concentrated on a small number of commercially widespread species—primarily *C. monogyna* Jacq. and *C. oxyacantha* L. For these species, the phenolic profile has been thoroughly characterized, modern extraction techniques have been optimized [9,10], and a broad spectrum of in vitro and in vivo biological activities has been established, including hepatoprotective effects—including amelioration of hepatic inflammation, fibrosis, and hepatocellular injury in experimental liver-disease models [11]. Toxicological evaluation, including assessment of potential teratogenic effects, has also been conducted [12]. These species thus constitute well-characterized models, while the vast majority of *Crataegus* species remains largely unexplored. Even among the studied species, phenolic profiles vary considerably: for example, *C. grayana* fruits and leaves show distinct, ripening-stage-dependent phenolic accumulation patterns [13], and marked interspecific differences in phenolic composition and associated nutraceutical potential have been reported across the berries, leaves, and flowers of several hawthorn species [14].

Over the past decade, a sustained trend towards phytochemical investigation of endemic and regional hawthorn species has emerged [15,16]. Among these is *Crataegus almaatensis* Pojark.—an endemic species native to the mountainous regions of Kazakhstan. Initial studies revealed that leaf extracts of *C. almaatensis* are characterized by markedly higher total phenolic content and superior antioxidant activity compared with extracts from flowers and fruits of the same species. Subsequent work led to the isolation and identification of three flavonoid glycosides—hyperoside, quercitrin, and afzelin—confirming the richness of this species in bioactive phenolics [17].

Building on these findings, our research group optimized an ultrasound-assisted extraction (UAE) protocol for *C. almaatensis* leaves, establishing conditions that maximized polyphenol recovery and demonstrating high in vitro antioxidant activity of the resulting extracts [18]. Subsequent LC-Q/TOF-MS metabolomic profiling characterized the composition of the optimized extract and established the predominance of chlorogenic acid and flavone-C-glycosides in its phenolic profile (unpublished data).

Despite these advances, research on *C. almaatensis* remains largely confined to extract-level analysis. Systematic chromatographic fractionation aimed at the isolation of individual metabolites in pure form and complete structural elucidation for this species has not previously been undertaken. In particular, minor and isomeric components of the phenolic profile remain uncharacterized, and structure-activity relationships for the identified constituents have not been established. Addressing this gap is essential for scientific validation of the species’ therapeutic potential and for the development of standardized preparations.

Beyond their pharmacological relevance, polyphenol-rich plant extracts are of growing interest as functional ingredients for the food industry, including confectionery manufacturing, where fortification with such extracts can offset the low nutritional value of traditional sugar-based products while enhancing their antioxidant capacity and health-promoting appeal [19]. Fruit-derived polyphenolic extracts in particular have been successfully incorporated into baked confectionery products such as cookies, improving phenolic content, antioxidant activity and enzyme-inhibitory properties without compromising sensory attractiveness [20]. Individual compound-level characterization of a candidate plant extract, as undertaken in the present study, therefore also provides a chemical basis for its rational application as a functional additive in confectionery formulations.

The present work is a direct continuation of our previous investigations [18] and aimed to: (i) perform multi-step chromatographic fractionation—column chromatography and preparative HPLC—of the optimized *C. almaatensis* leaf extract; (ii) isolate secondary metabolites as individual pure form; (iii) unequivocally elucidate the structures of the isolated aglycones and define the substitution pattern of the isolated compounds using a combination of modern spectroscopic methods—UPLC-ESI-QTOF-MS/MS, and one- and two-dimensional NMR spectroscopy (HSQC, HMBC).

## 2. Materials and Methods

### 2.1. Plant Material

Aerial parts (leaves) of *C. almaatensis* Pojark. were collected at the fruit-ripening stage from mature, healthy trees growing wild in the mountainous area of Ile-Alatau National Park (Almaty region, Kazakhstan; approximately 1700–2330 m a.s.l., Kimasar Gorge area) between 5–24 August 2025, as part of research project IRN AP23489874. Leaves were pooled from 3–4 individual trees located at least 20–30 m apart, and material collected on different days was combined into a single composite sample prior to extraction. Field identification was based on morphological characters consistent with the known distribution of *C. almaatensis* in the Trans-Ili (Ile-Alatau) mountain range, where the species has previously been formally identified and vouchered by the Institute of Botany and Phytointroduction (Almaty) from nearby gorges of the same range (Medeu, Alma-Arasan) [17]. Botanical identification of the collected material was confirmed by the Institute of Botany and Phytointroduction (Almaty; Ministry of Ecology and Natural Resources of the Republic of Kazakhstan), identification certificate No. 01-05/453, dated 17 August 2026, issued for a confirmatory specimen collected from the same species population in the Trans-Ili Alatau range (Alma-Arasan gorge) in August 2026. Freshly collected leaves were dried at ambient temperature in the shade and stored in paper bags prior to extraction.

### 2.2. Chemicals and Reagents

Methanol, n-hexane, dichloromethane (DCM), and ethyl acetate (EtOAc) of analytical grade were used for extraction and chromatographic separation. Silica gel (particle size 40–63 µm) was employed for column chromatography. Deuterated dimethyl sulfoxide (DMSO-d_6_) was used as the NMR solvent. All solvents and reagents were obtained from commercial suppliers and used without further purification unless otherwise stated.

### 2.3. Extraction and Isolation

#### 2.3.1. Preparation of the Crude Methanolic Extract

**Figure 1:**
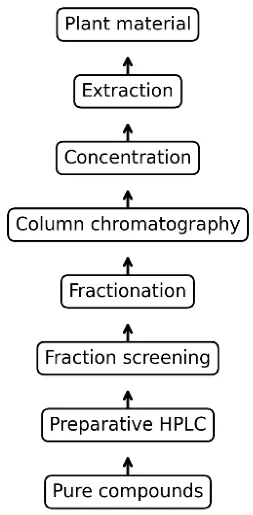
Schematic representation of the isolation and purification of biologically active compounds from *C. almaatensis* leaves.

Air-dried leaves of *C. almaatensis* (330 g total) were subjected to ultrasound-assisted extraction (UAE) with methanol (three extraction cycles of 30 min each, ~40 kHz, 30 °C), maintaining a solid-to-solvent ratio of approximately 1:10 (*w*/*v*). After each cycle, the extract was filtered through filter paper, and the filtrate was concentrated under reduced pressure at 40–45 °C by rotary evaporation. The combined extracts yielded a dark crude methanolic residue (69 g). The overall isolation and purification workflow is summarized in Figure 1.

#### 2.3.2. Liquid–Liquid Partitioning

The crude extract (69 g) was suspended in 500 mL distilled water and subjected to sequential liquid–liquid partitioning. The aqueous suspension was extracted three times with equal volumes of n-hexane to remove non-polar constituents. Subsequently, the aqueous layer was extracted three times with ethyl acetate (EtOAc; 500 mL per cycle) at 45 °C for 25–30 min under continuous stirring (75 rpm) and reduced pressure (160 hPa). The combined EtOAc fractions were concentrated under reduced pressure at 45 °C to yield the ethyl acetate-soluble fraction.

#### 2.3.3. Column Chromatography

The EtOAc fraction was adsorbed onto silica gel (used at approximately 1.5 times the mass of the EtOAc fraction) and loaded onto a silica gel column pre-packed with n-hexane. Gradient elution was performed with increasing polarity: 100% n-hexane; n-hexane/DCM mixtures (2–90% DCM); 100% DCM; DCM/methanol mixtures (1–10% MeOH); and 20% MeOH in DCM. Collected sub-fractions were monitored by TLC and combined to yield 22 pooled fractions. Each fraction was concentrated to dryness and filtered through a 0.45 µm membrane filter prior to further purification.

#### 2.3.4. Preparative HPLC Fractionation

Fraction 22 was further purified by preparative HPLC (Agilent 1260 Infinity (Agilent Technologies, Santa Clara, CA, USA), HYPERSIL GOLD column (Thermo Fisher Scientific, Waltham, MA, USA), 250 × 10 mm; mobile phase: 10% MeCN in H_2_O to 100% MeCN over 20 min; flow rate: 3.0 mL min^−1^; column temperature: 45 °C) to yield compounds **4** (tR 7.30 min, 130.9 mg), **5** (tR 9.78 min, 23.8 mg), **1** (tR 10.30 min, 17.5 mg), **3** (tR 13.59 min, 4.8 mg), and **2** (tR 14.88 min, 4.1 mg).

Homogeneity of each isolated compound was assessed by the appearance of a single symmetrical peak under the analytical LC-MS conditions described in Section 2.4 and by the absence of extraneous signals in the corresponding 1D NMR spectra (Supplementary Figures S1–S21). Analytical purity (% peak area) was 95.40% (**1**), 94.94% (**2**), 98.57% (**3**), 96.43% (**4**), and 96.08% (**5**); the corresponding purity chromatograms are provided as Figures S22–S26 (Supplementary Materials).

### 2.4. Analytical LC-MS Monitoring of Fractions

The Agilent low-resolution LC-MS system described below was used exclusively for routine analytical monitoring of column and preparative-HPLC fractions during purification (peak tracking and preliminary nominal-mass dereplication); it did not serve as the basis for structure elucidation. Accurate mass measurements used for molecular formula determination of the isolated compounds were obtained separately on the Waters QToF system described in Section 2.5. Analytical HPLC-MS was performed using an Agilent 1200 UPLC system coupled to an Agilent 1946D mass spectrometer (Agilent Technologies, Santa Clara, CA, USA) on an Eclipse XDB-C8 column (4.6 × 150 mm, 5 µm). Mobile phase: (A) acetonitrile, (B) 0.05% formic acid in water; gradient: 0–15 min 10–100% A, 15–20 min 100% A, 20–20.5 min 100–10% A, 20.5–25 min 10% A; flow rate 1.0 mL min^−1^; column temperature 28 °C. MS parameters: mass range *m*/*z* 100–1000; drying gas (N_2_) 35 °C, 12 L min^−1^.

### 2.5. Structure Elucidation

One-dimensional (1H, 13C) and two-dimensional (HSQC, HMBC, 1H-1H COSY) NMR experiments were performed on a Bruker Avance III spectrometer (Bruker, Billerica, MA, USA) (500 MHz). Chemical shifts (δ) are reported in ppm relative to the residual solvent signal of DMSO-d_6_ (δ_H_ 2.50, δ_C_ 39.52). Coupling constants (J) are given in Hz. Accurate-mass measurements were performed using a quadrupole time-of-flight (QTOF) mass spectrometer coupled with an electrospray ionization (ESI) source (Waters ACQUITY UPLC I-Class/Xevo G2 XS QToF system, Manchester, UK). Optical rotations [α]^25^,_D_ were recorded, where sufficient sample quantity was available, on an Anton Paar MCP 200 Modular Circular Polarimeter (Anton Paar, Graz, Austria) in a 100 × 2 mm cell.

### 2.6. ABTS Radical Scavenging Assay

ABTS [2,2′-azino-bis (3-ethylbenzothiazoline-6-sulfonic acid)] was dissolved in ultrapure water to prepare a 7 mmol/L stock solution. Potassium persulfate (K_2_S_2_O_8_) was separately dissolved in ultrapure water to prepare a 140 mmol/L stock solution. Five millilitres of the 7 mmol/L ABTS solution were mixed thoroughly with 88 µL of the 140 mmol/L potassium persulfate solution to obtain a final potassium persulfate concentration of 2.45 mmol/L. The resulting ABTS working solution was incubated in the dark at room temperature for 12–16 h. Before use, the ABTS working solution was diluted with ethanol to an absorbance of 0.700 ± 0.020 at 734 nm. Sample solutions were prepared in DMSO at eight concentrations (4, 8, 16, 32, 64, 128, 256, and 512 µg/mL), and vitamin C (Vc), used as the positive control, was prepared at five concentrations (1, 20, 30, 50, and 80 µg/mL). Each reaction was performed in a 96-well plate with a final volume of 200 µL, comprising 100 µL of sample (or Vc) solution and 100 µL of the ABTS working solution; plates were shaken and incubated at room temperature for 6 min, and the absorbance of each well was then measured at 734 nm. IC_50_ values were determined by fitting the log concentration–response curves using a four-parameter variable-slope nonlinear regression model in GraphPad Prism (version 11; GraphPad Software, Boston, MA, USA). All samples were tested in triplicate (three independent measurements) to ensure the accuracy and reliability of the results.

### 2.7. DPPH Radical Scavenging Assay

2,2-Diphenyl-1-picrylhydrazyl (DPPH) was dissolved in ethanol to obtain a 1.0 × 10^−3^ mol/L stock solution, which was stored at 4 °C and diluted with ethanol to 0.2 mmol/L prior to use. Sample solutions were prepared in DMSO at eight concentrations (4, 8, 16, 32, 64, 128, 256, and 512 µg/mL), and vitamin C (Vc), used as the positive control, was prepared at five concentrations (1, 20, 30, 50, and 80 µg/mL). Each reaction was performed in a 96-well plate with a final volume of 200 µL, comprising 100 µL of sample (or Vc) solution and 100 µL of the DPPH working solution; plates were mixed thoroughly and incubated in the dark at room temperature for 30 min, and the absorbance of each well was then measured at 517 nm. IC_50_ values for both the ABTS and DPPH assays were determined by fitting the log concentration–response curves using a four-parameter variable-slope nonlinear regression model in GraphPad Prism (version 11; GraphPad Software, Boston, MA, USA). Each sample was tested in triplicate to ensure experimental reliability.

## 3. Results

### 3.1. Compound Isolation and Structural Elucidation

**Figure 2:**
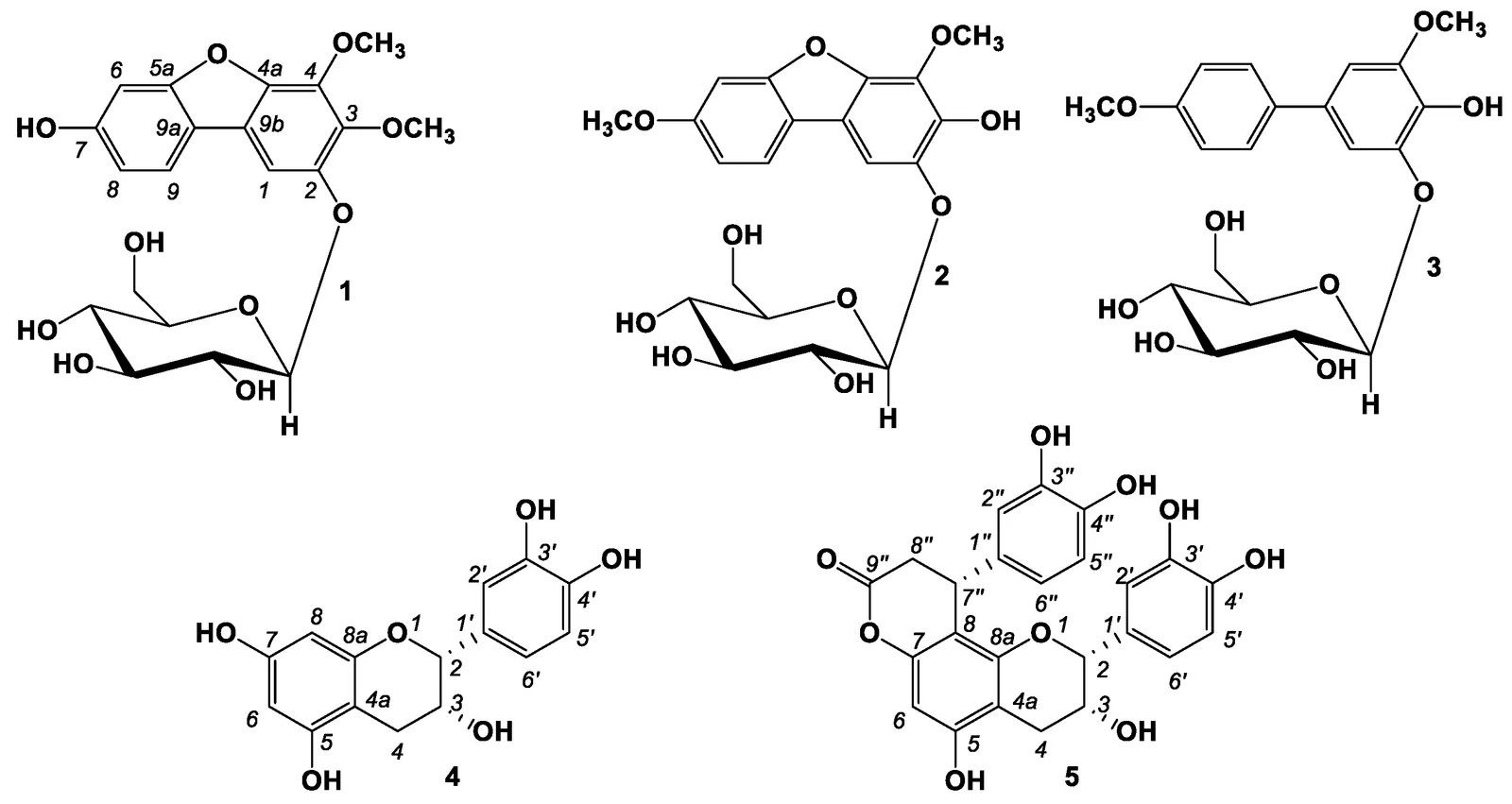
Chemical structures of compounds isolated from *C. almaatensis* leaves.

Multi-step chromatographic fractionation of the methanol extract of *C. almaatensis* leaves afforded five individual phenolic compounds, designated compounds **1**–**5** (Figure 2). The structures of compounds **1** and **2** were elucidated by comprehensive HR-ESI-MS and 1D/2D NMR spectroscopic analysis. The structures of compounds **3**–**5** were identified by comparison of spectroscopic data with literature data.

### 3.2. Compound ***1***: 3-O-Methylcrataegusnoside G

**Figure 3:**
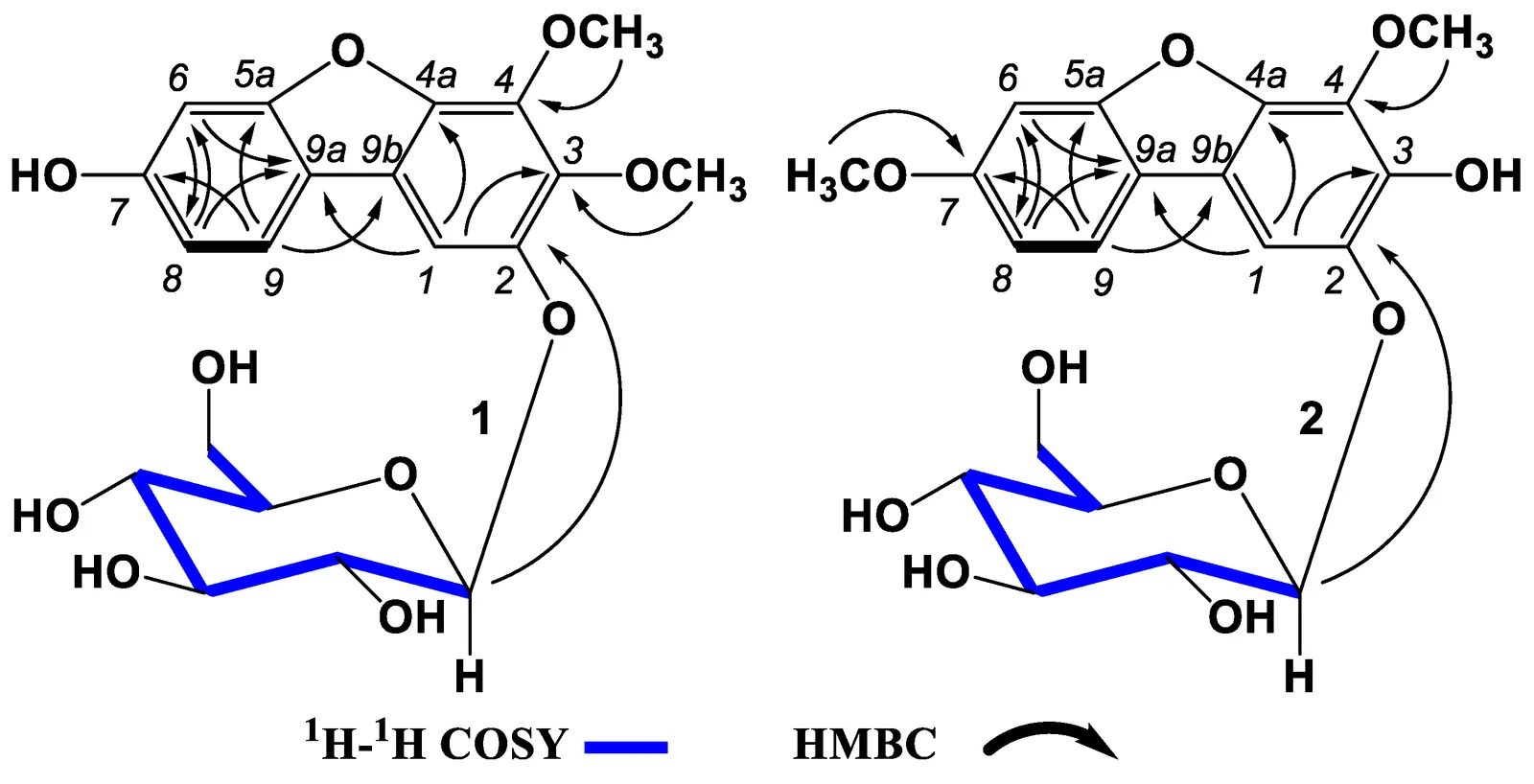
Key ^1^H-^1^H COSY and HMBC correlations for compounds **1** and **2**.

Compound **1** was isolated as a light-yellow amorphous solid (yield 17.5 mg). Its molecular formula, C_20_H_22_O_10_, was established by HR-ESI-MS (*m*/*z* 421.1143 [M − H]^−^, calcd for C_20_H_21_O_10_^−^: 421.1140), indicating ten degrees of unsaturation. The ^1^H and ^13^C NMR data (Table 1) were consistent with a penta-substituted, dibenzofuran-type biphenyl skeleton (H-1, H-6, H-8, H-9 and the corresponding aromatic carbons) bearing two methoxy groups and a β-D-glucopyranosyl unit attached at C-2 (anomeric signal δ_H_ 4.92, d, J = 7.5 Hz, H-1′; δ_C_ 101.8, C-1′). In the HMBC spectrum, the HMBC correlations from H-1 to C-3, C-4a, and C-9a; H-9 to C-5a and C-7; and H-6 to C-8 and C-9a revealed the presence of a biphenyl moiety. The HMBC correlations from H-OCH_3_-3 to C-3 and H-OCH_3_-4 to C-4 defined the location of two methoxy groups. The HMBC correlation from H-1′ to C-2 confirmed the attachment of the sugar moiety at C-2 (Figure 3). The sugar moiety was characterized as glucose by comparing NMR data with previously reported analogues [21]. Thus, compound **1** was established as 7-hydroxy-3,4-dimethoxydibenzofuran 2-O-β-glucopyranoside and named 3-O-methyl-crataegusnoside G.

### 3.3. Compound ***2***: 7-O-Methylcrataegusnoside G

Compound **2** was isolated as a light-yellow amorphous powder (yield 4.1 mg). Its molecular formula was established as C_20_H_22_O_10_ by HR-ESI-MS (*m*/*z* 421.1138 [M − H]^−^, calcd for C_20_H_21_O_10_^−^: 421.1140), indicating ten degrees of unsaturation. The ^1^H and ^13^C NMR data of compound **2** (Table 1) closely resembled those of compound **1**, indicating the same dibenzofuran-glucopyranoside skeleton. The key differences were confined to the methoxy-bearing ring: in compound **1** the two OCH3 signals correlated (HMBC) to C-3 and C-4, whereas in compound **2** they correlated to C-4 and C-7, and the aromatic proton shifts in the AMX system (H-6, H-8, H-9) were correspondingly displaced (δ_H_ 7.27, 6.94, 7.77 vs. 7.00, 6.81, 7.74 in **1**). This regiochemical shift in the HMBC correlation pattern, rather than a simple duplication of compound **1**, indicates that **1** and **2** are positional isomers differing in the relative placement of the two methoxy groups on the dibenzofuran ring, not the same compound. As in compound **1**, in the HMBC spectrum, the HMBC correlations from H-1 to C-3, C-4a, and C-9a; H-9 to C-5a and C-7; and H-6 to C-8 and C-9a revealed the presence of a biphenyl moiety. The HMBC correlations from H-OCH_3_-4 to C-4 and H-OCH_3_-7 to C-7 defined the location of two methoxy groups. The HMBC correlation from H-1′ to C-2 in the HMBC spectrum confirmed the location of β-pyranose at C-2 (Figure 3). By comparing the NMR data with those of compound **1**, the sugar moiety was identified to be glucose. Thus, compound **2** was established as 3-hydroxy-4,7-dimethoxydibenzofuran 2-O-β-glucopyranoside and named 7-O-methylcrataegusnoside G.

The aglycone structures of compounds **1** and **2**, including the position of the two methoxy substituents, were established unambiguously from the HMBC correlations described above. The identity of the sugar moiety as β-D-glucopyranose was assigned by comparison of its ^1^H/^13^C chemical-shift pattern and anomeric coupling constant (J = 7.5 Hz, consistent with a β-configured pyranose) with literature data for glucopyranosyl-substituted phenolics; this NMR-based assignment does not by itself exclude another hexose (e.g., galactose) with a similar substitution pattern. Confirmation by acid hydrolysis and comparison of the released sugar with an authentic standard was beyond the scope of the present study and is planned as follow-up work. Optical rotation data for compounds **1** and **2** could not be obtained owing to the limited quantity isolated; this is noted explicitly as a limitation in the Conclusions Section.

### 3.4. Compound ***3***: Kakispyrol

Compound **3** was isolated as a light-yellow amorphous solid (yield 4.8 mg). HR-ESI-MS showed *m*/*z* 407.1342 [M − H]^−^ (calcd for C_20_H_23_O_9_: 407.1342), corresponding to the molecular formula C_20_H_24_O_9_ and nine degrees of unsaturation. The NMR spectra revealed a diarylpropanoid glycoside framework. By comparing the NMR data with previously reported values, compound **3** was identified as kakispyrol [21]. Its presence in *C. almaatensis* expands the list of known natural sources of this compound and reflects the diversity of lignan constituents in this species.

### 3.5. Compound ***4***: (−)-Epicatechin

Compound **4** was isolated as a light-yellow amorphous solid (yield 130.9 mg). Its molecular formula was established as C_15_H_14_O_6_ based on the HR-ESI-MS (*m*/*z* 289.0714 [M − H]^−^, calcd for C_15_H_13_O_6_^−^: *m*/*z* 289.0718), indicating nine degrees of unsaturation. By comparing the NMR data (Table 2) with previously reported values, compound **4** was identified as (−)-epicatechin [22].

### 3.6. Compound ***5***—Cinchonain Ia

Compound **5** was isolated as a yellow amorphous solid (yield 23.8 mg). HR-ESI-MS showed a pseudomolecular ion at *m*/*z* 451.1040 [M − H]^−^ (calcd for C_24_H_19_O_9_: 451.1035), corresponding to the molecular formula C_24_H_20_O_9_ (15 degrees of unsaturation), indicative of a highly unsaturated polyphenolic structure. The ^1^H and ^13^C NMR spectra revealed a flavolignan skeleton comprising an epicatechin unit linked to a phenylpropanoid moiety. By comparing the NMR data (Table 2) with previously reported values, compound **5** was identified as cinchonain Ia.

Cinchonains are rare flavolignans first isolated from *Cinchona* species. They are characterized by a hybrid structure resulting from the condensation of a flavonoid and a phenylpropanoid moiety [23,24]. The identification of cinchonain Ia from *C. almaatensis* is a particularly noteworthy result, as compounds of this class are encountered far less frequently than simple flavonoids.

### 3.7. General Characterization of the Phenolic Profile

In total, five phenolic compounds belonging to three chemical classes were isolated and structurally characterized from the leaves of *Crataegus almaatensis*: flavanols (epicatechin), flavolignans (cinchonain Ia), and diaryl ether-type phenolic glycosides. This profile is consistent with the broader chemical diversity documented across the genus *Crataegus* and adds to the still-limited phytochemical characterization of *C. almaatensis* specifically. Of particular note is the isolation of cinchonain Ia—a rare flavolignan whose presence in hawthorn species is infrequently reported—and the identification of kakispyrol, extending the list of known natural sources of this compound. These results provide the structural basis for systematic investigation of structure-activity relationships of the bioactive constituents of *C. almaatensis* leaves.

Compounds **1** and **2** extend, rather than duplicate, the previously reported phenolic profile of *C. almaatensis*, which had until now been limited to the flavonoid glycosides hyperoside, quercitrin, and afzelin [17]. Dibenzofuran-type diaryl ether glycosides of this kind have not, to our knowledge, been previously reported from any *Crataegus* species, in contrast to the flavan-3-ol and proanthocyanidin-type constituents (e.g., epicatechin, cinchonain-type flavolignans) that dominate the phytochemical literature of the genus [3,4,23,24]. Their occurrence in *C. almaatensis* adds a metabolite class not previously reported from this species to its phytochemical profile; whether this reflects a broader distinction from other *Crataegus* species remains to be established and will require comparative phytochemical screening across the genus together with confirmation against authentic standards.

### 3.8. Antioxidant Activity

The antioxidant capacities of the isolated compounds are summarized in Table 3 (concentration–response curves are provided as Figures S27 (ABTS) and S28 (DPPH) in the Supplementary Materials). Vitamin C (Vc) was employed as a positive control for comparative analysis in this study. In the ABTS assay, the IC50 values ranged from 3.25 μg/mL to 23.72 μg/mL. Compound **4** exhibited the best antioxidant activity with an IC50 of 3.25 μg/mL, while compound **2** showed the lowest activity at 23.72 μg/mL. Vitamin C, used as the positive control, showed an IC_50_ value of 55.32 μg/mL. In the DPPH assay, compound **5** exhibited the strongest radical-scavenging activity, with an IC_50_ value of 32.05 μg/mL, which was notably lower than, and therefore more active than, the vitamin C positive control (53.13 μg/mL). Compound **3** (kakispyrol) was not evaluated in either assay owing to the limited quantity obtained upon isolation (4.8 mg). Overall, the tested samples exhibited distinct antioxidant capacities in ABTS and DPPH radical scavenging assays. It should be noted that ABTS and DPPH are both single-electron-transfer-based chemical assays and do not capture mechanisms such as metal chelation, lipid-peroxidation inhibition, or activity in a cellular context; the relative ranking of the compounds reported here should therefore be interpreted as an indicative chemical antioxidant profile rather than a direct measure of biological antioxidant potential, and complementary assays, such as FRAP, ORAC, and cell-based models, are required to validate these findings.

## 4. Conclusions

A systematic phytochemical investigation of *C. almaatensis* Pojark leaves led to the isolation and structural characterization of five phenolic compounds: 3-O-methylcrataegusnoside G (1), 7-O-methylcrataegusnoside G (2), kakispyrol (3), (−)-epicatechin (4), and cinchonain Ia (5), belonging to three chemical classes (dibenzofuran-type diaryl ether glycosides, a flavolignan, and a flavanol). Structures were established from HR-ESI-MS and 1D/2D NMR data (Table 1 and Table 2); for compounds **1** and **2**, the aglycone substitution pattern was defined unambiguously by HMBC, while the assignment of the sugar moiety as glucopyranose rests on NMR chemical-shift comparison rather than hydrolysis with an authentic standard. Compounds **1**, **2**, **4**, and **5** exhibited measurable ABTS and DPPH radical-scavenging activity, whereas compound **3** was not evaluated owing to the limited quantity available.

This study has several limitations. The identity of the hexose unit in compounds **1** and 2 was assigned spectroscopically and has not been confirmed by acid hydrolysis and comparison with an authentic sugar standard. Analytical purity data (percentage peak area, purity chromatograms) for the isolated compounds are reported in the Supplementary Materials but were not used to correct the reported yields. Antioxidant activity was assessed using only the ABTS and DPPH chemical assays, which do not fully represent biological antioxidant mechanisms. Compound **3** was isolated in insufficient quantity for antioxidant testing.

The results expand knowledge of the secondary metabolite profile of *C. almaatensis* and identify this species as a source of structurally diverse phenolic compounds, including cinchonain Ia—a flavolignan infrequently reported in the genus *Crataegus*—and dibenzofuran-type diaryl ether glycosides not previously reported from this genus. Future work should prioritize definitive structural confirmation of compounds **1** and **2** against authentic standards, evaluation of their biological activities beyond chemical antioxidant assays, and broader metabolite profiling of *C. almaatensis*.

## Figures and Tables

**Table 1:** NMR spectroscopic data of compounds **1**–**3** (1H, 500 MHz; 13C, 125 MHz; DMSO-d_6_).

Pos.	1		2		3	
	δ_C_	δ_H_ (*J*, Hz)	δ_C_	δ_H_ (*J*, Hz)	δ_C_	δ_H_ (*J*, Hz)
1	101.3	7.48 (^1^H, s)	102.6	7.53 (^1^H, s)	105.3	6.89 (^1^H, d, 2.0)
2	148.2		143.6		148.4	
3	140.3		139.2		135.6	
4	138.4		133.0		146.1	
4a	142.3		144.1		107.8	7.07 (^1^H, d, 2.0)
5a	157.5		156.8		127.4	7.57 (2H, d, 9.0)
6	98.2	7.00 (^1^H, d, 2.0)	96.8	7.27 (^1^H, d, 2.0)	114.2	6.94 (^1^H, d, 9.0)
7	157.4		158.7		158.4	
8	111.8	6.81 (^1^H, dd, 8.5, 2.0)	110.9	6.94 (^1^H, dd, 8.5, 2.0)	114.2	6.94 (^1^H, d, 9.0)
9	121.0	7.74 (^1^H, d, 8.5)	120.4	7.77 (^1^H, d, 8.5)	127.4	7.57 (^1^H, d, 9.0)
9a	115.5		117.3		132.8	
9b	119.9		114.9		130.4	
3-OCH_3_	61.3	3.83 (3H, s)				
4-OCH_3_	60.8	4.07 (3H, s)	60.5	4.01 (3H, s)	56.1	3.83 (3H, s)
7-OCH_3_			55.7	3.84 (3H, s)	55.2	3.77 (3H, s)
1′	101.8	4.92 (^1^H, d, 7.5)	103.6	4.71 (^1^H, d, 7.5)	102.7	4.72 (^1^H, m)
2′	73.4	3.32 (^1^H, m)	73.4	3.35 (^1^H, m)	73.5	3.28 (^1^H, m)
3′	77.2	3.37 (^1^H, m)	77.4	3.37 (^1^H, m)	77.4	3.30 (^1^H, m)
4′	69.9	3.19 (^1^H, t, 9.5)	69.9	3.19 (^1^H, t, 9.5)	70.2	3.15 (^1^H, m)
5′	76.9	3.31 (^1^H, m)	75.9	3.30 (^1^H, m)	76.0	3.30 (^1^H, m)
6′	60.9	3.74 (^1^H, d, 12.0); 3.48 (^1^H, dd, 12.0, 6.0)	60.9	3.78 (^1^H, d, 12.0); 3.51 (^1^H, dd, 12.0, 6.0)	61.0	3.41 (2H, m)

**Table 2:** NMR spectroscopic data of compounds **4** and **5** (1H, 500 MHz; 13C, 125 MHz; DMSO-d_6_).

Pos.	4 [22]		5 [23]	
	**δ_C_**	**δ_H_ (J, Hz)**	**δ_C_**	**δ_H_ (J, Hz)**
2	78.1	4.73 (^1^H, brs)	78.1	4.73 (^1^H, brs)
3	64.9	4.00 (^1^H, brs)	64.1	3.98 (^1^H, brs)
4	28.2	2.67 (^1^H, dd, 16.5, 4.5); 2.47 (^1^H, dd, 16.5, 3.5)	28.4	2.67 (^1^H, dd, 16.5, 4.5); 2.47 (^1^H, dd, 16.5, 3.5)
4a	98.5		103.9	
5	156.5		155.6	
6	95.1	5.71 (^1^H, d, 2.5)	94.9	6.19 (^1^H, s)
7	156.2		151.8	
8	94.1	5.89 (^1^H, d, 2.5)	103.9	
8a	155.8		150.1	
1′	130.6		130.3	
2′	114.8	6.89 (^1^H, d, 1.5)	114.3	6.88 (^1^H, d, 2.0)
3′	144.5		145.1	
4′	144.5		144.0	
5′	114.9	6.67 (^1^H, d, 8.0)	115.6	6.67 (^1^H, d, 8.0)
6′	118.0	6.65 (^1^H, dd, 8.0, 1.5)	117.4	6.64 (^1^H, dd, 8.0, 2.0)
1″			133.1	
2″			114.9	6.40 (^1^H, d, 2.0)
3″			144.4	
4″			144.6	
5″			115.6	6.56 (^1^H, d, 8.0)
6″			117.4	6.34 (^1^H, dd, 8.0, 2.0)
7″			33.2	4.40 (^1^H, d, 7.5)
8″			37.5	3.13 (^1^H, dd, 15.5, 7.0); 2.78 (^1^H, 15.5, 1.5)
9″			168.0	

**Table 3:** Antioxidant activity of compounds **1**–**5** (IC50, μg/mL).

	1	2	3	4	5	Vc
ABTS	12.58	23.72	-	3.25	8.77	55.32
DPPH	78.98	104.10	-	57.98	32.05	53.13

## Data Availability

The raw data supporting the conclusions of this article will be made available by the authors upon request.

## Supplementary Materials

The following supporting information can be downloaded at https://www.mdpi.com/article/10.3390/antiox15091149/s1. Figures S1–S21: HR-ESI-MS and 1D/2D NMR spectra (^1^H, ^13^C, HSQC, COSY, HMBC) of compounds **1**–**5**; Figures S22–S26: analytical purity chromatograms of compounds **1**–**5**; Figures S27 and S28: ABTS and DPPH concentration–response curves, respectively.

## Abbreviations

The following abbreviations are used in this manuscript:

NMR	Nuclear Magnetic Resonance
HR-ESI-QTOF-MS	High-Resolution Electrospray Ionization Quadrupole Time-of-Flight Mass Spectrometry
HSQC	Heteronuclear Single Quantum Coherence
HMBC	Heteronuclear Multiple Bond Correlation
COSY	Correlation Spectroscopy
DMSO-d_6_	Deuterated Dimethyl Sulfoxide
UAE	Ultrasound-Assisted Extraction
