# Supplementary material for: Phytochemical Investigation of Crataegus almaatensis Leaves: Isolation, Structural Characterization, and Antioxidant Activity of Flavonoids, Flavolignans, and Phenolic Glycosides
Source: Antioxidants (Basel). 2026 Sep 10;15(9):1149. doi: 10.3390/antiox15091149 (PMC13603708; doi:10.3390/antiox15091149)
Supplement: Supplementary file 1 [file antioxidants-15-01149-s001.zip › antioxidants-4478211-supplementary.pdf]

Table of Contents

Figure S1. HR-ESI-MS spectrum for **1** 3

Figure S2. ^{1}H NMR spectrum (500 MHz, DMSO-d_6) of **1** 3

Figure S3. ^{13}C NMR spectrum (125 MHz, DMSO-d_6) of **1** 4

Figure S4. HSQC spectrum (500 MHz, DMSO-d_6) of **1** 4

Figure S5. ^{1}H -^{1}H COSY spectrum (500 MHz, DMSO-d_6) of **1** 5

Figure S6. HMBC spectrum (500 MHz, DMSO-d_6) of **1** 5

Figure S7. HR-ESI-MS spectrum for **2** 6

Figure S8. ^{1}H NMR spectrum (500 MHz, DMSO-d_6) of **2** 6

Figure S9. ^{13}C NMR spectrum (125 MHz, DMSO-d_6) of **2** 7

Figure S10. HSQC spectrum (500 MHz, DMSO-d_6) of **2** 7

Figure S11. ^{1}H -^{1}H COSY spectrum (500MHz, DMSO-d_6) of **2** 8

Figure S12. HMBC spectrum (500MHz, DMSO) of **2** 8

Figure S13. HR-ESI-MS spectrum for **3** 9

Figure S14. ^{1}H NMR spectrum (500 MHz, DMSO-d_6) of **3** 9

Figure S15. ^{13}C NMR spectrum (125 MHz, DMSO-d_6) of **3** 10

Figure S16. HR-ESI-MS spectrum for **4** 10

Figure S17. ^{1}H NMR spectrum (500 MHz, DMSO-d_6) of **4** 11

Figure S18. ^{13}C NMR spectrum (125 MHz, DMSO-d_6) of **4** 11

Figure S19. HR-ESI-MS spectrum for **5** 12

Figure S20. ^{1}H NMR spectrum (500 MHz, DMSO-d_6) of **5** 12

Figure S21. ^{13}C NMR spectrum (125 MHz, DMSO-d_6) of **5** 13

Figure S22. HPLC profile of 1 13

Figure S23. HPLC profile of 2 14

Figure S24. HPLC profile of 3 14

Figure S25. HPLC profile of 4 .... **15**

Figure S26. HPLC profile of 5 .... **15**

Figure S27. ABTS radical-scavenging concentration-response curves of compounds 1, 2, 4, 5 .... **16**

Figure S28. DPPH radical-scavenging concentration-response curves of compounds 1, 2, 4, 5 .... **17**

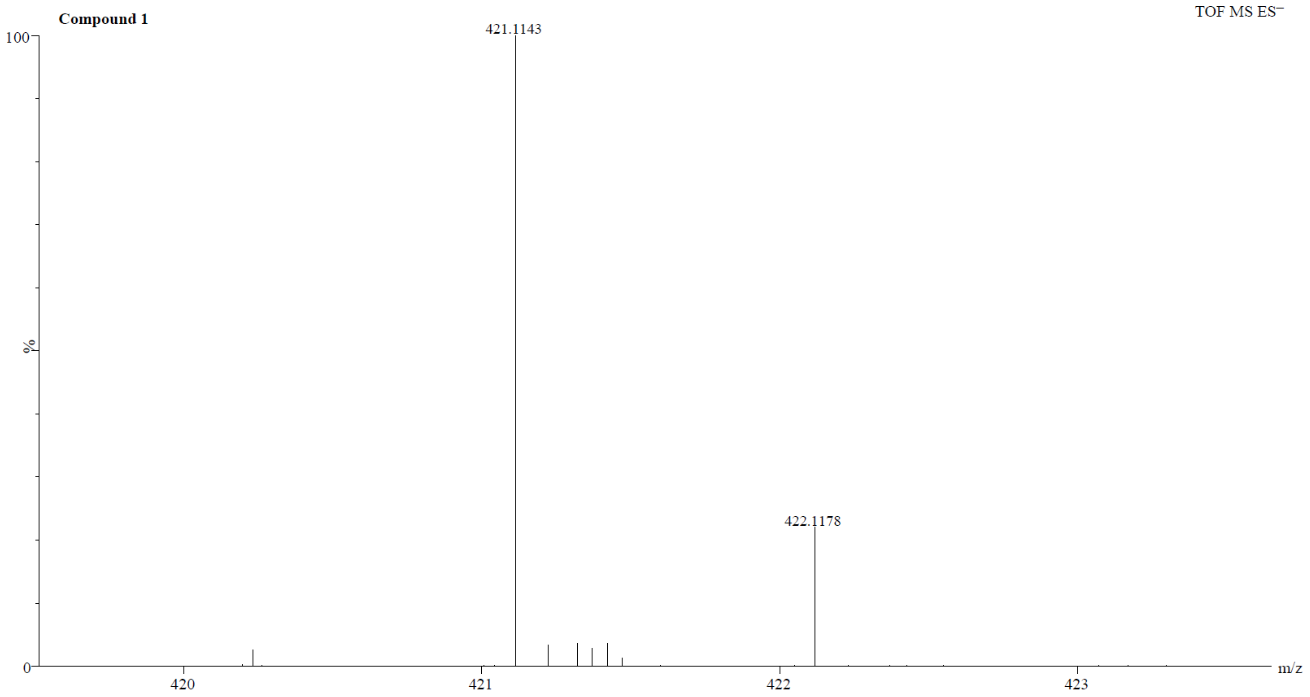

Figure S1. HR-ESI-MS spectrum for compound **1**

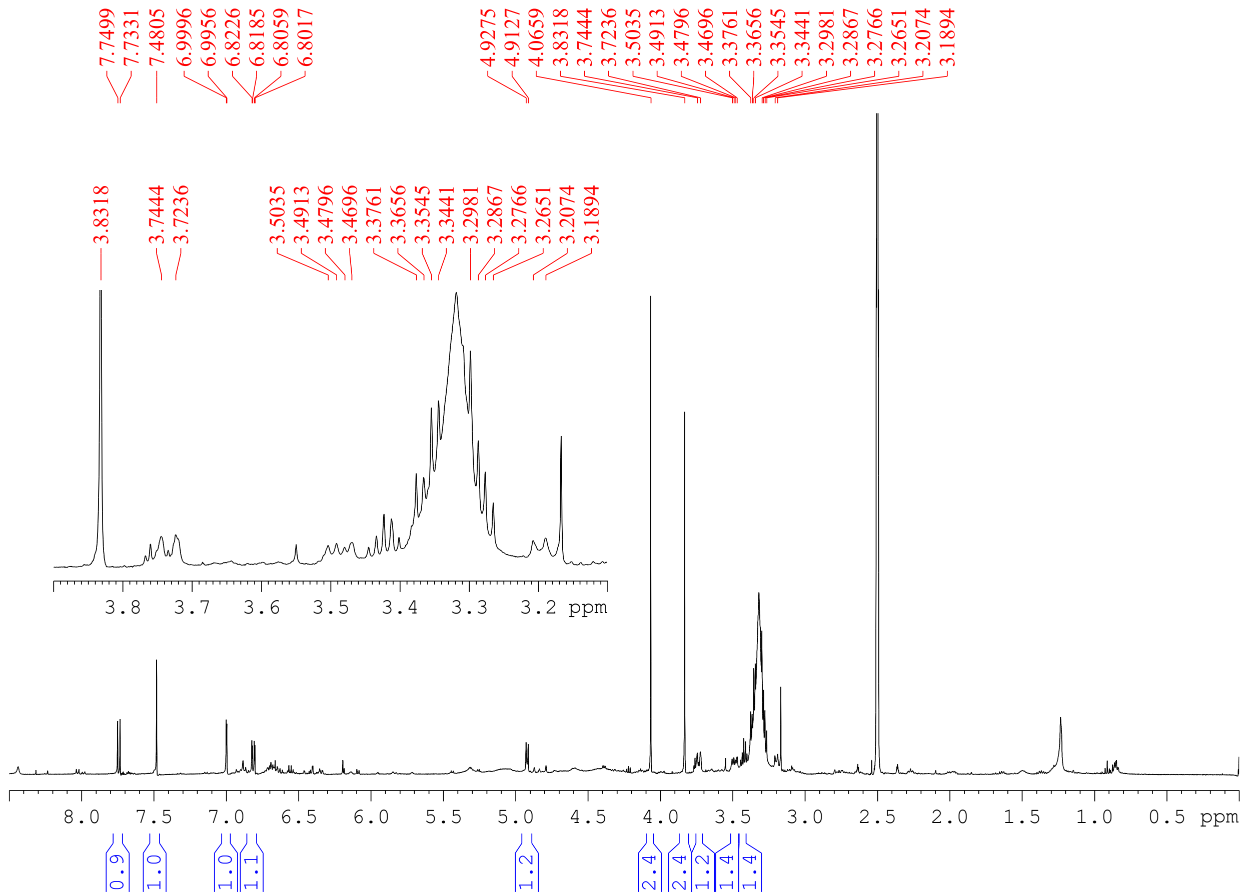

Figure S2. ^{1}H NMR spectrum (500 MHz, DMSO-d_6) of **1**

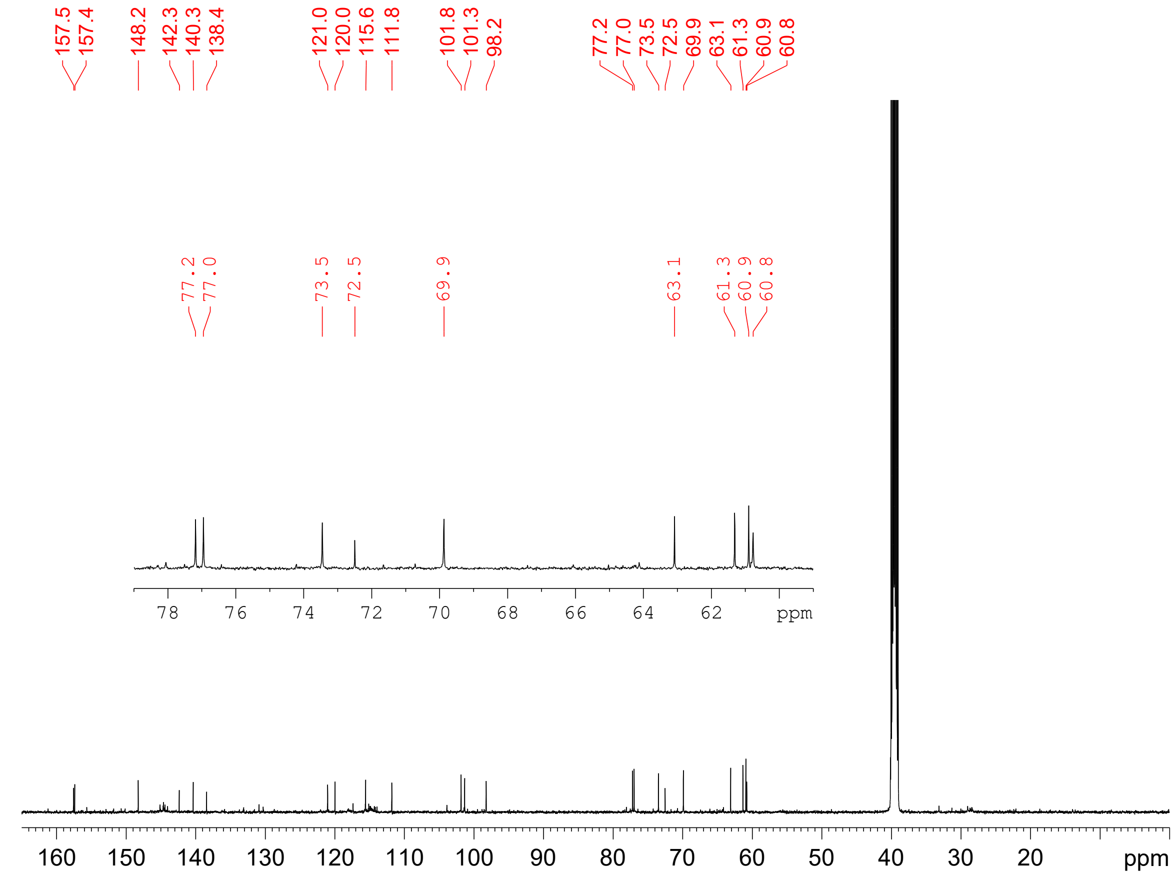

Figure S3. ^{13}C NMR spectrum (125 MHz, DMSO-d_6) of **1**

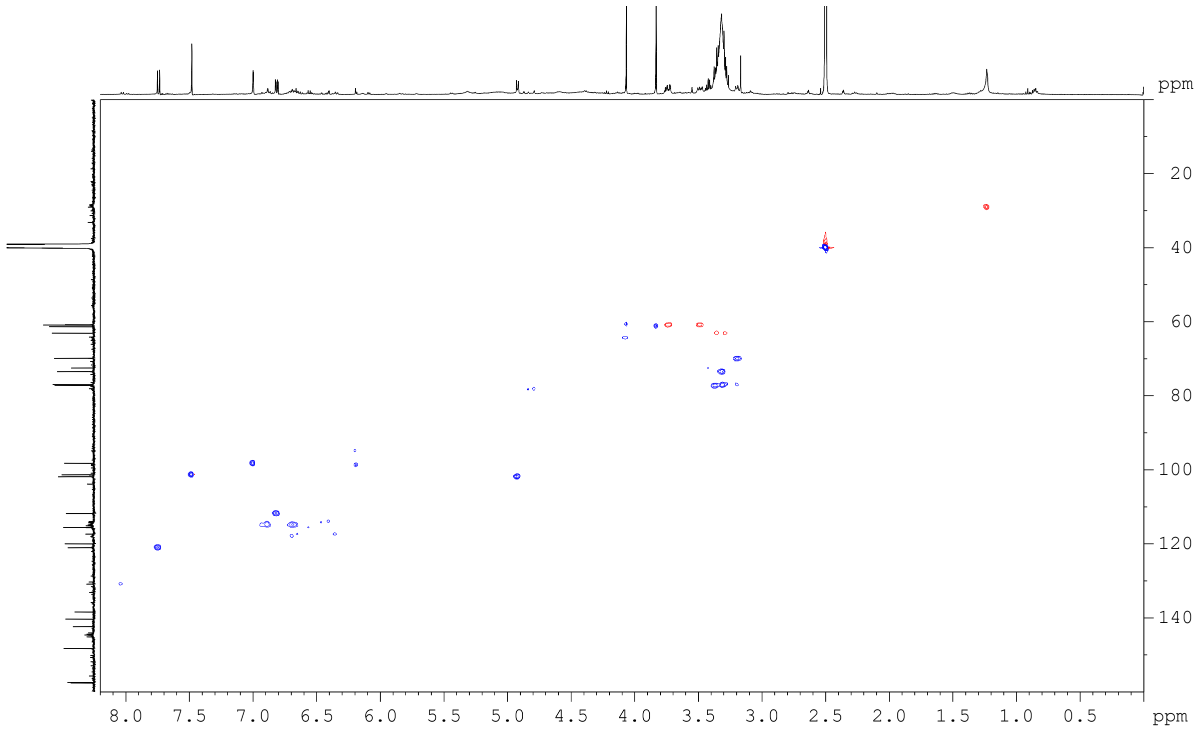

Figure S4. HSQC spectrum (500 MHz, DMSO-d_6) of **1**

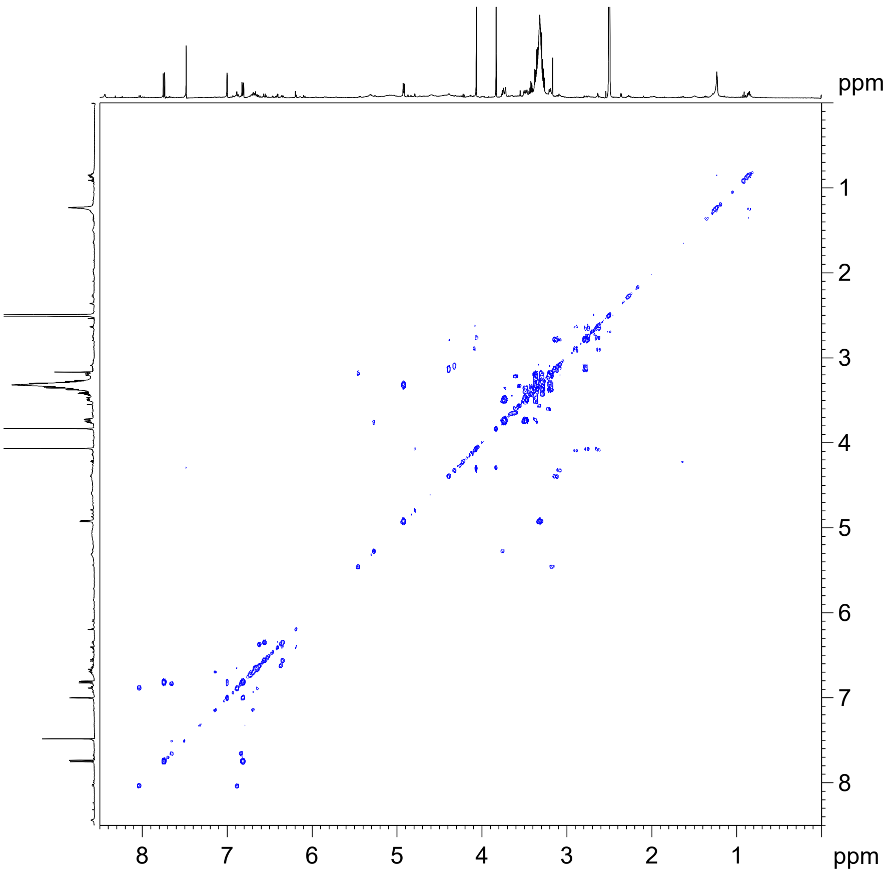

Figure S5. ^{1}H -^{1}H COSY spectrum (500 MHz, DMSO-d_6) of **1**

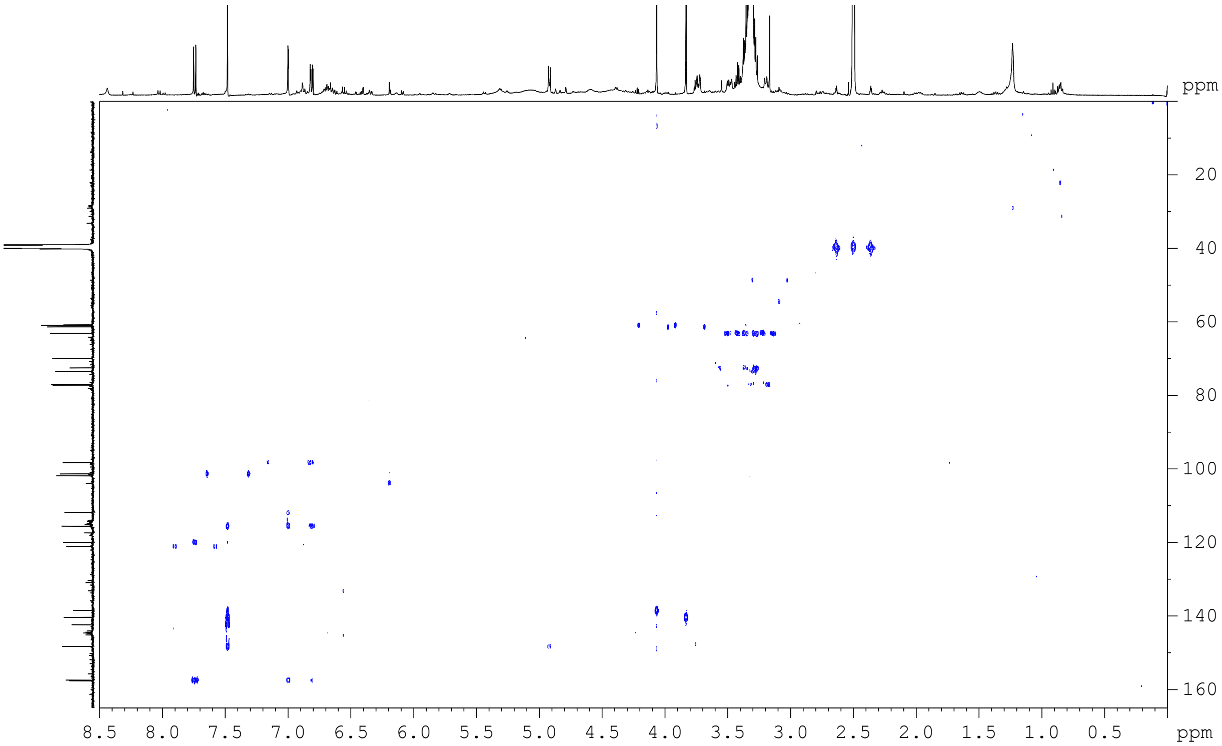

Figure S6. HMBC spectrum (500 MHz, DMSO-d_6) of **1**

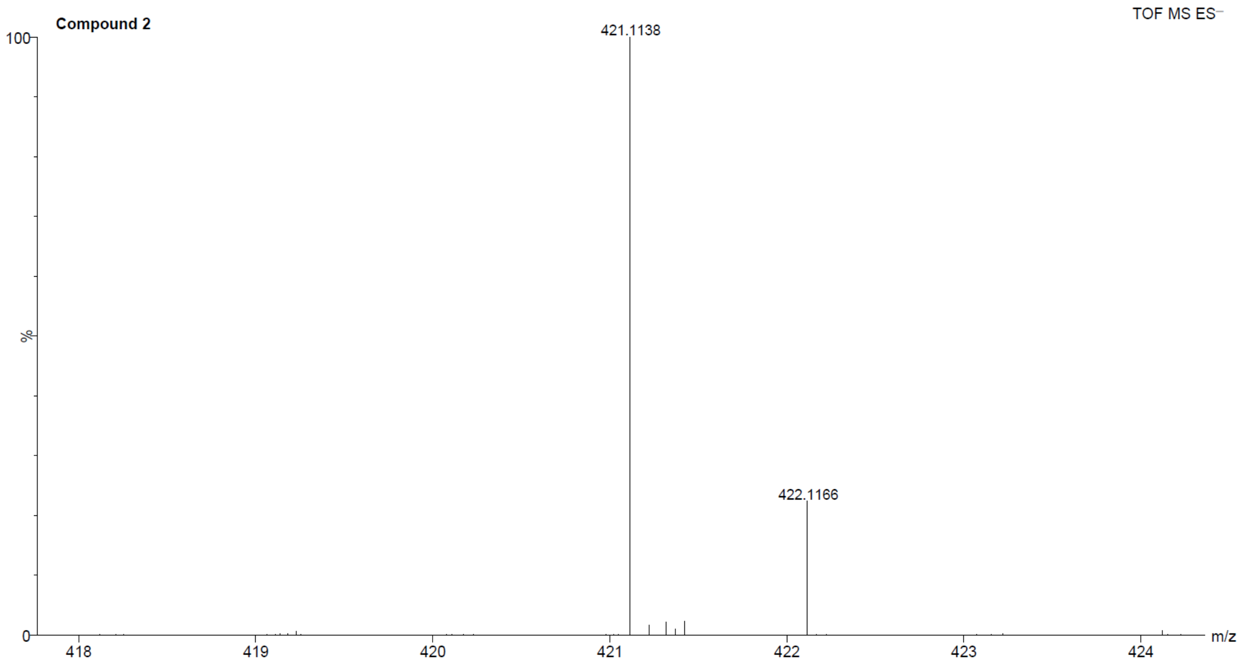

Figure S7. HR-ESI-MS spectrum for **2**

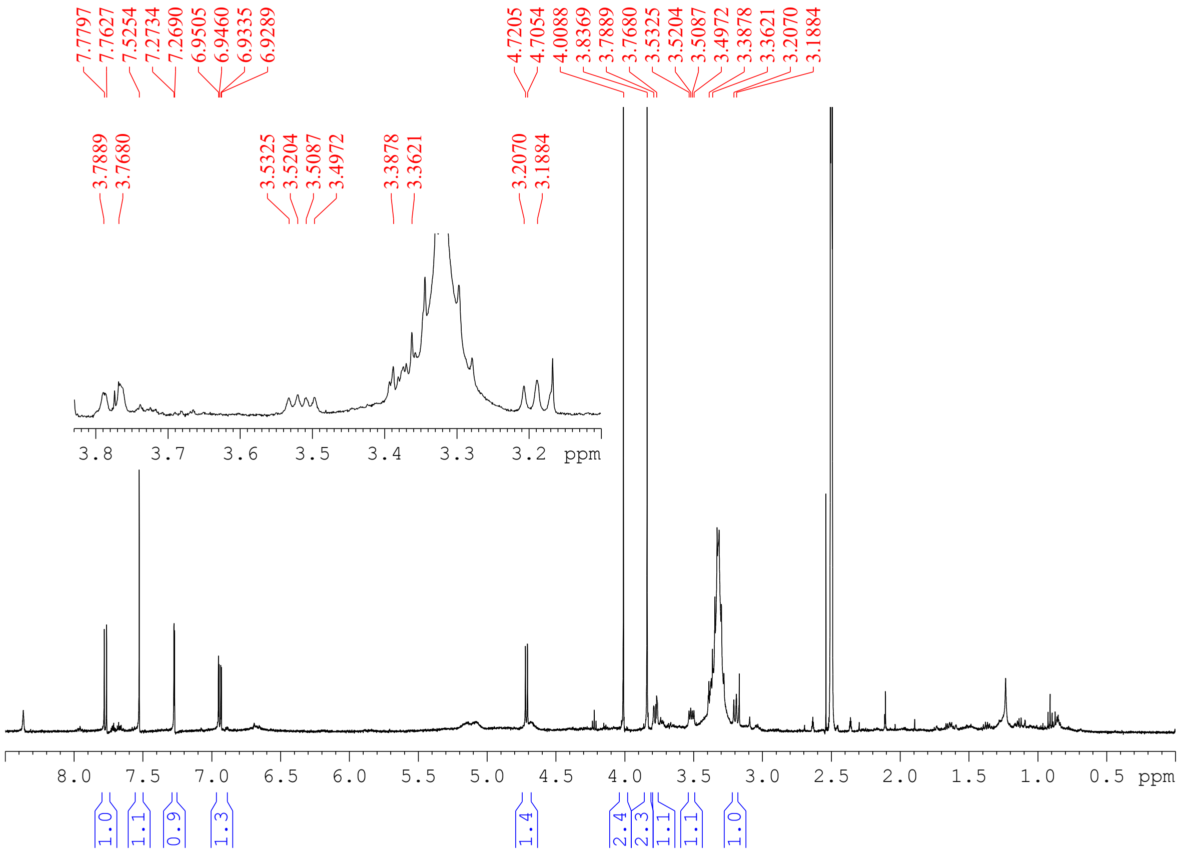

Figure S8. ^{1}H NMR spectrum (500 MHz, DMSO-d_6) of **2**

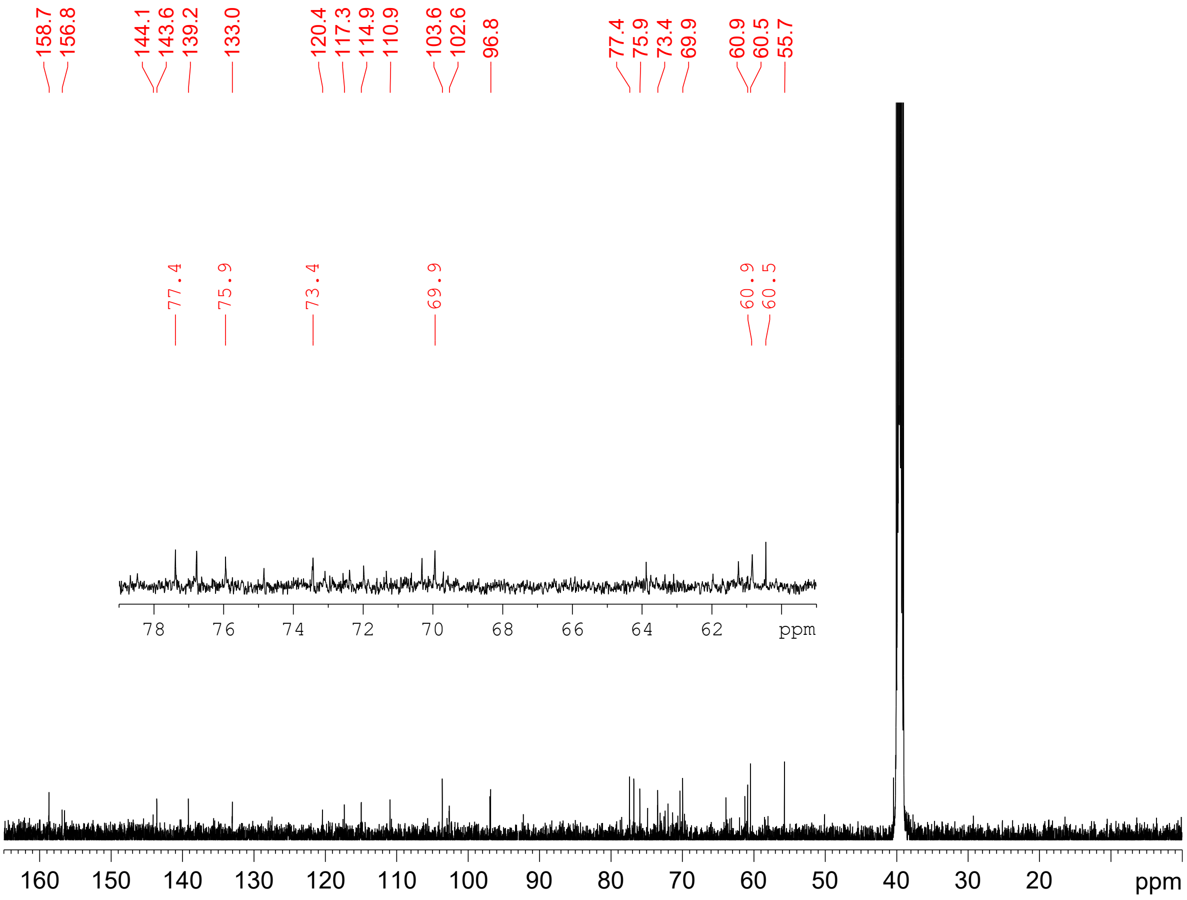

Figure S9. ^{13}C NMR spectrum (125 MHz, DMSO-d_6) of **2**

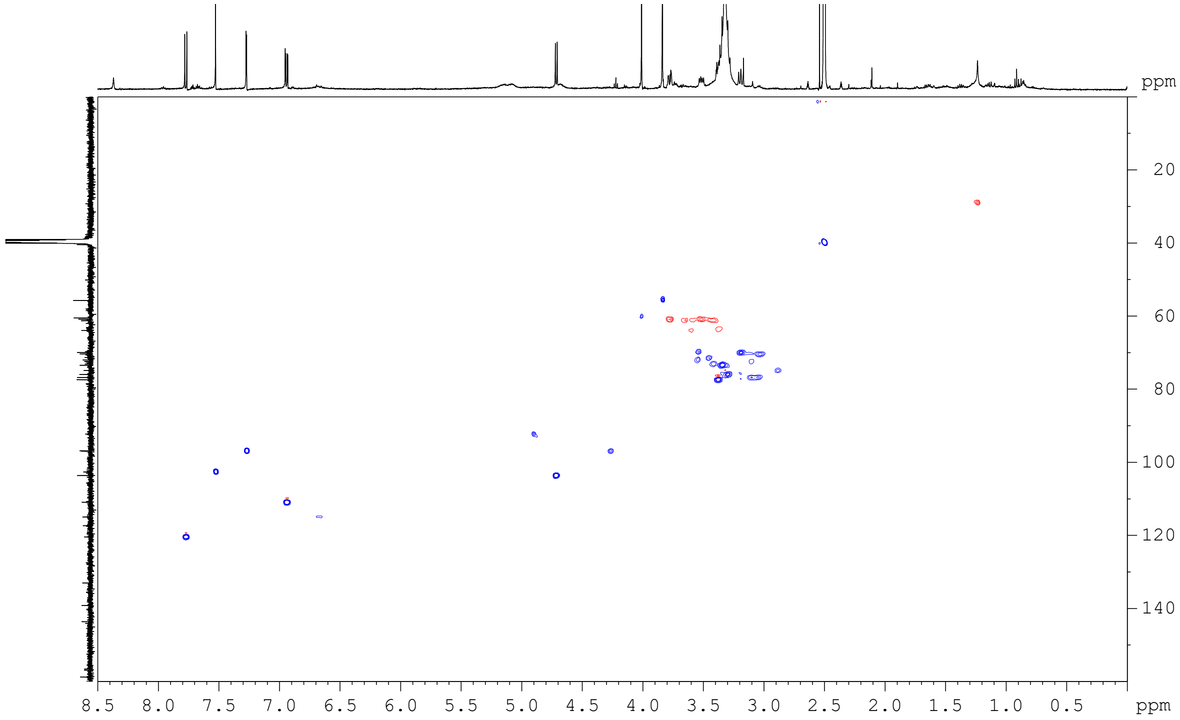

Figure S10. HSQC spectrum (500 MHz, DMSO-d_6) of **2**

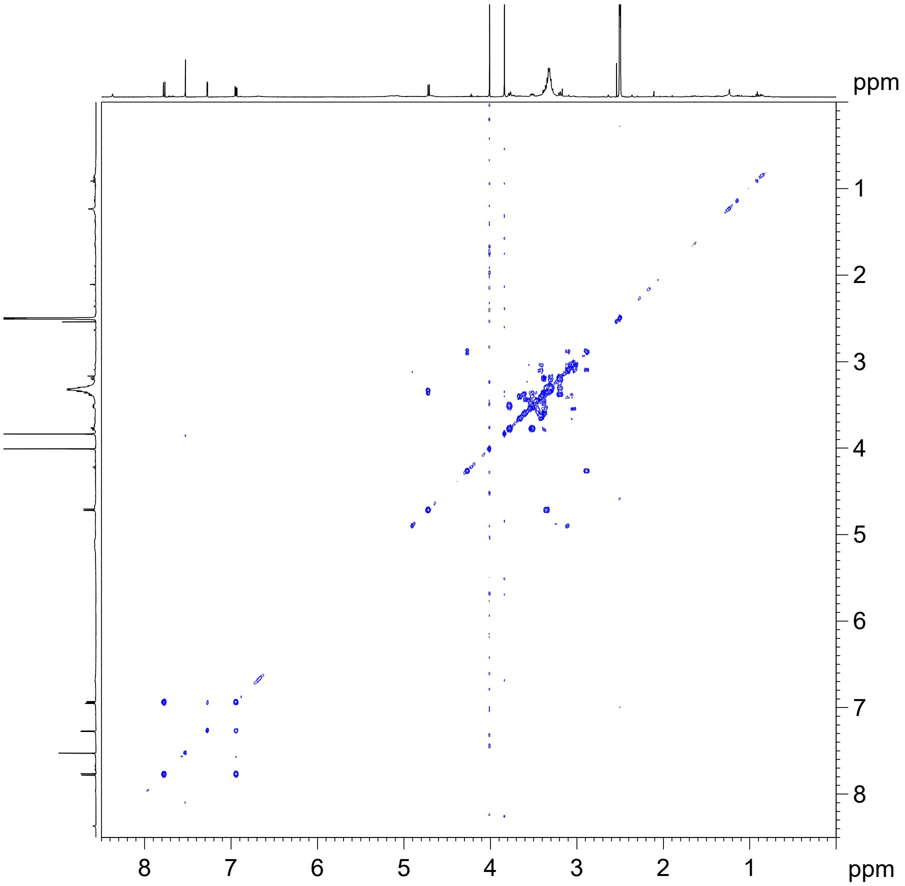

Figure S11. ^{1}H -^{1}H COSY spectrum (500MHz, DMSO-*d*6) of **2**

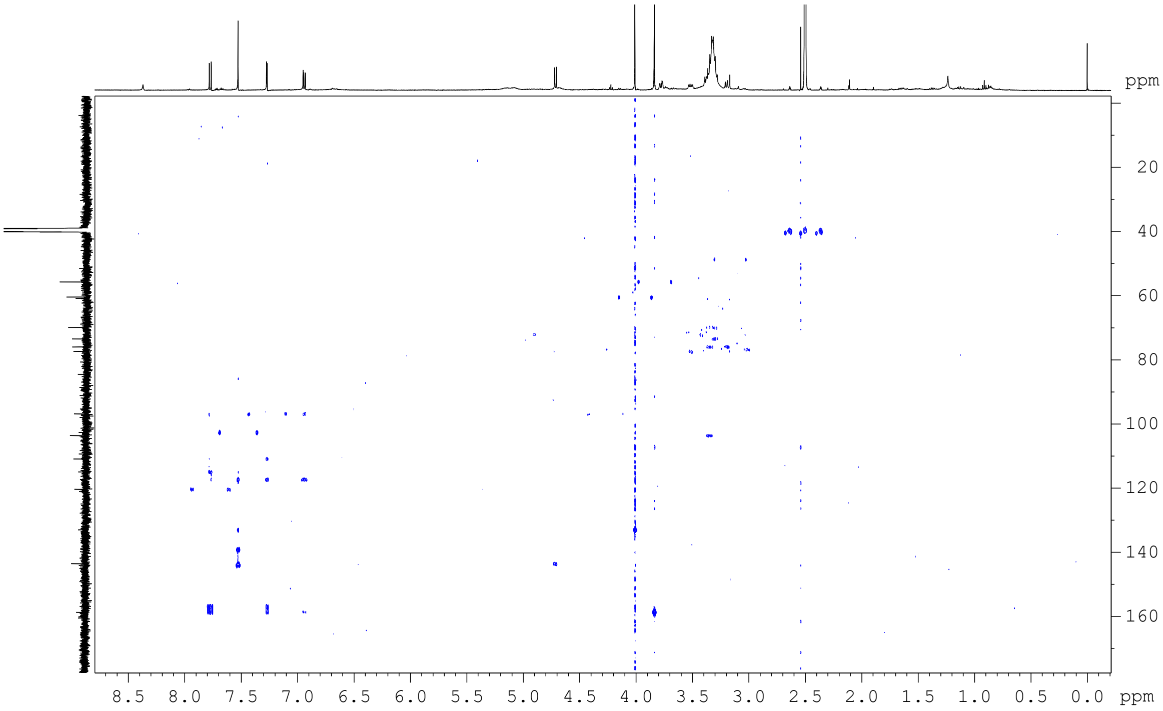

Figure S12. HMBC spectrum (500 MHz, DMSO-*d*6) of **2**

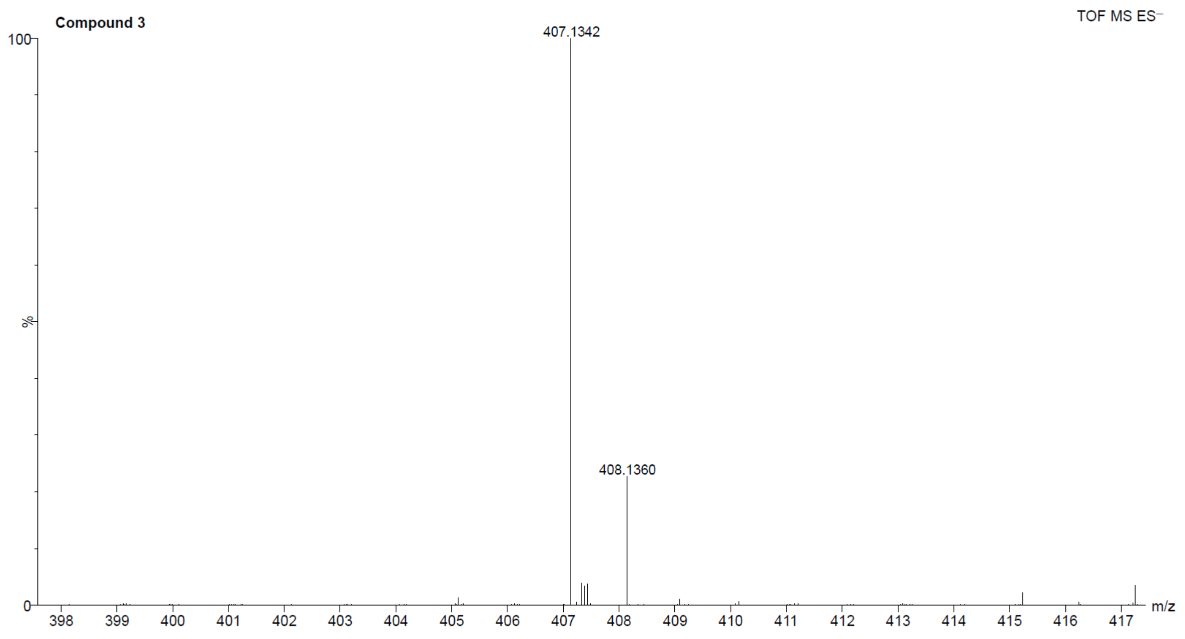

Figure S13. HR-ESI-MS spectrum for **3**

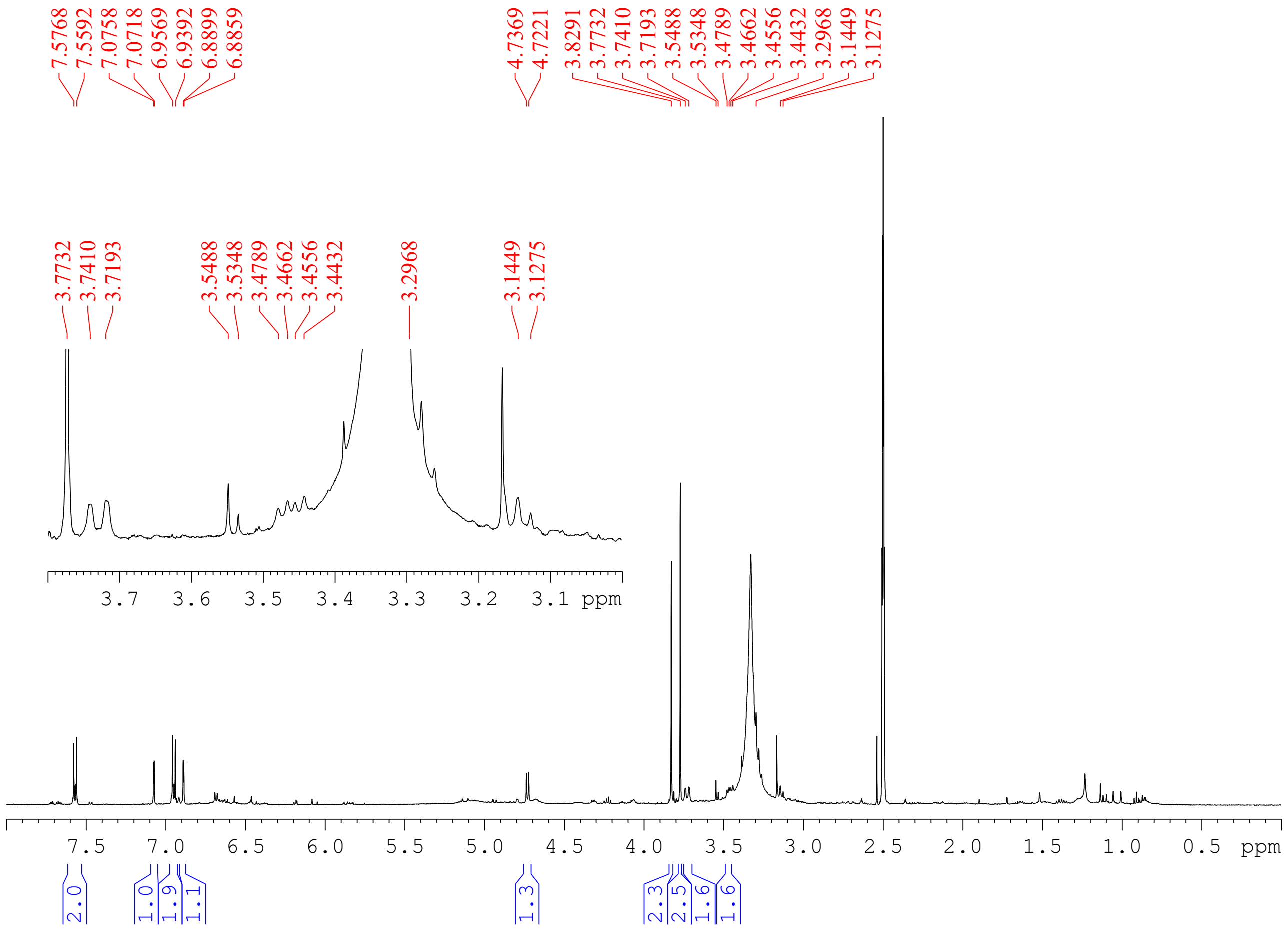

Figure S14. ^{1}H NMR spectrum (500 MHz, DMSO-d_6) of **3**

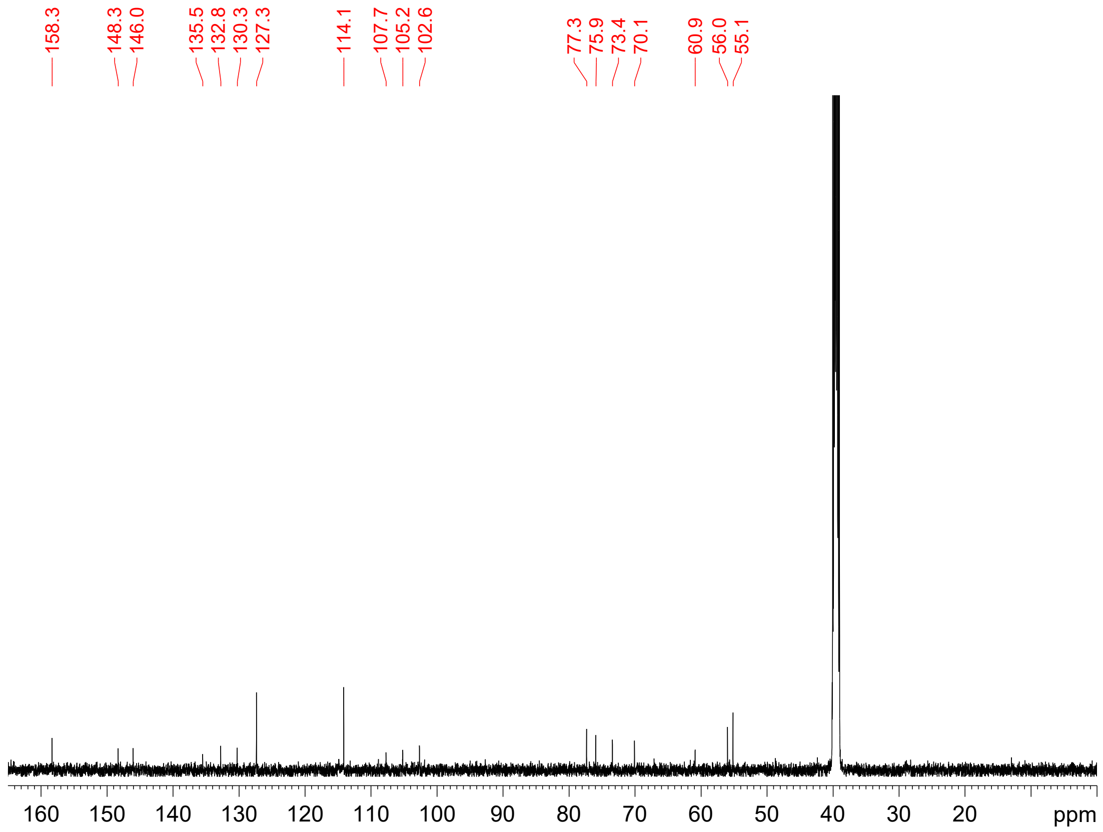

Figure S15. ^{13}C NMR spectrum (125 MHz, DMSO-d_6) of **3**

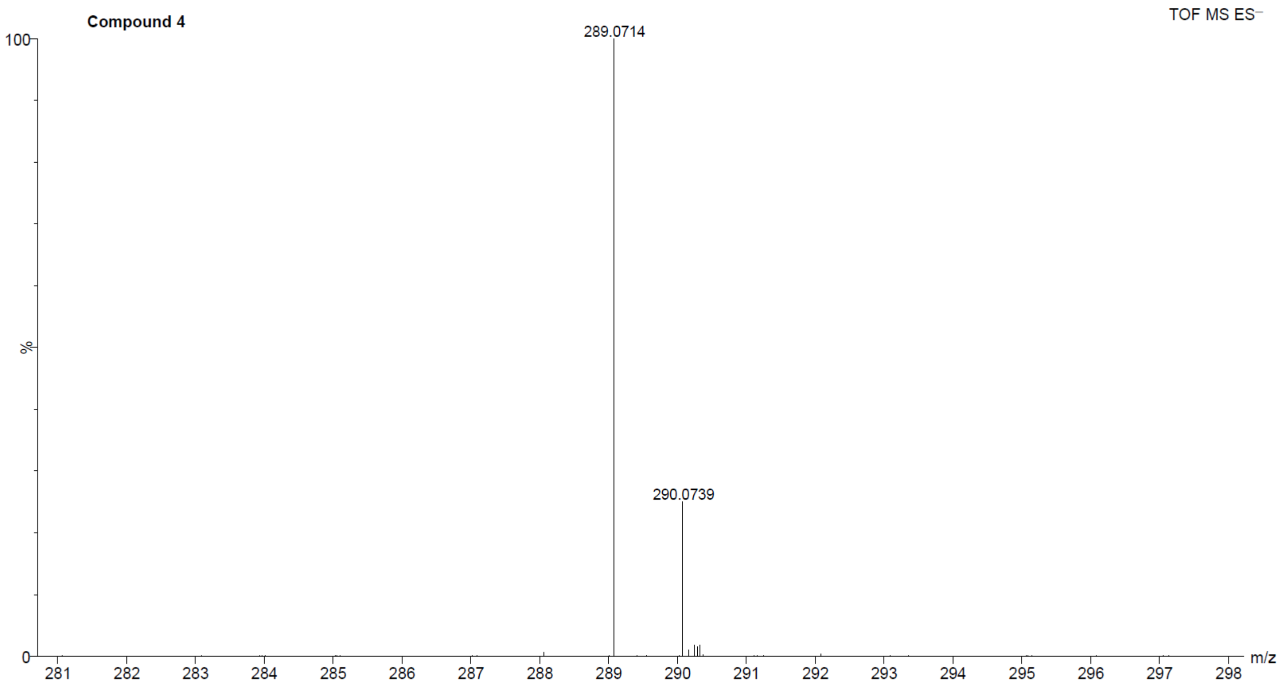

Figure S16. HR-ESI-MS spectrum for **4**

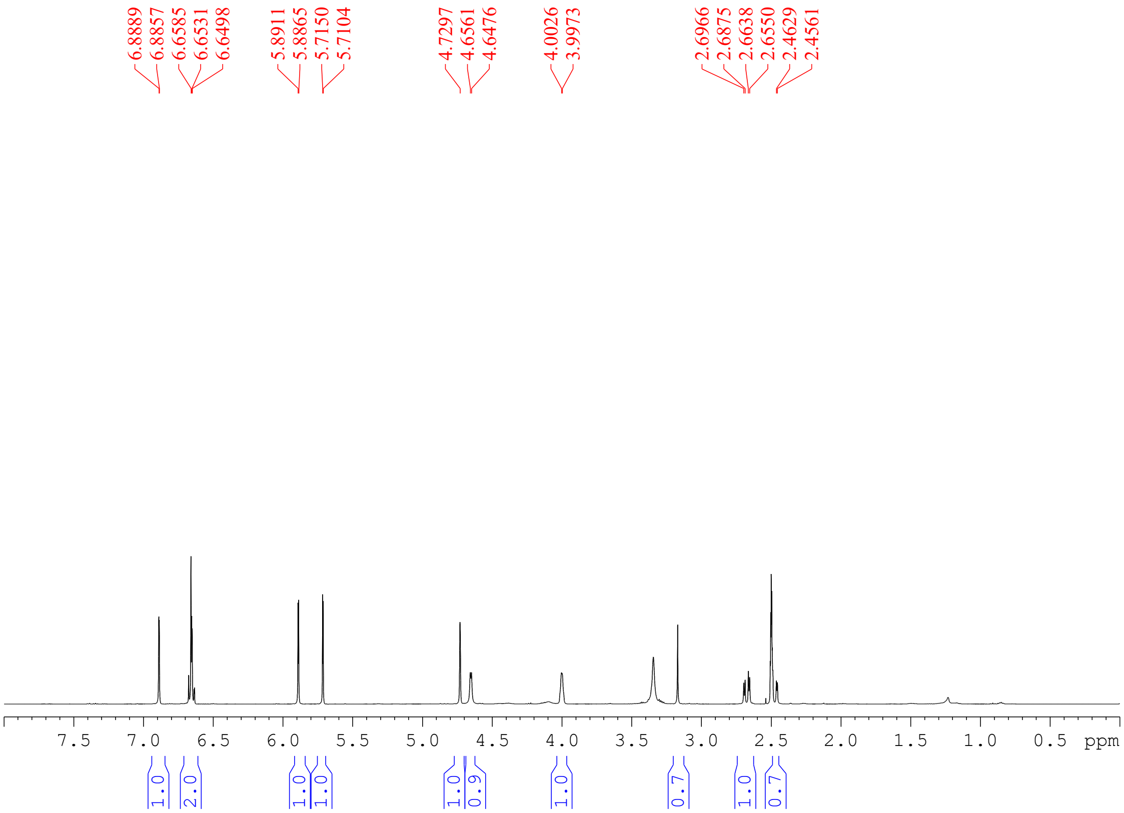

Figure S17. ^{1}H NMR spectrum (500 MHz, DMSO-d_6) of **4**

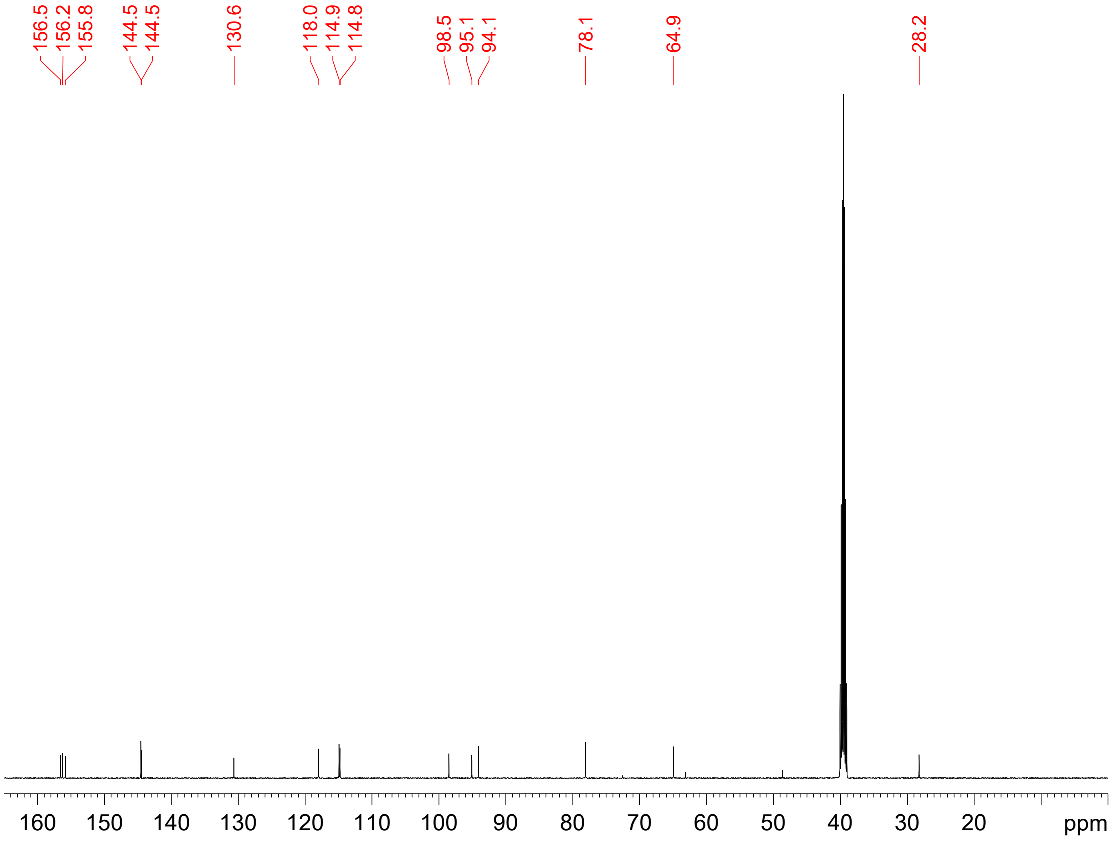

Figure S18. ^{13}C NMR spectrum (125 MHz, DMSO-d_6) of **4**

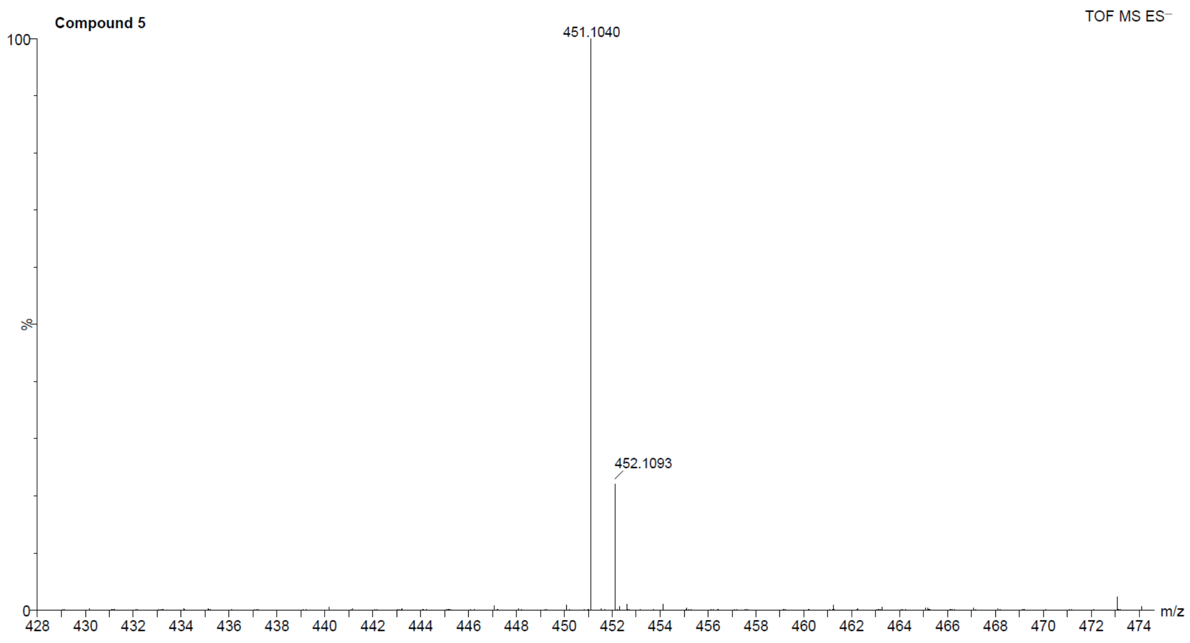

Figure S19. HR-ESI-MS spectrum for **5**

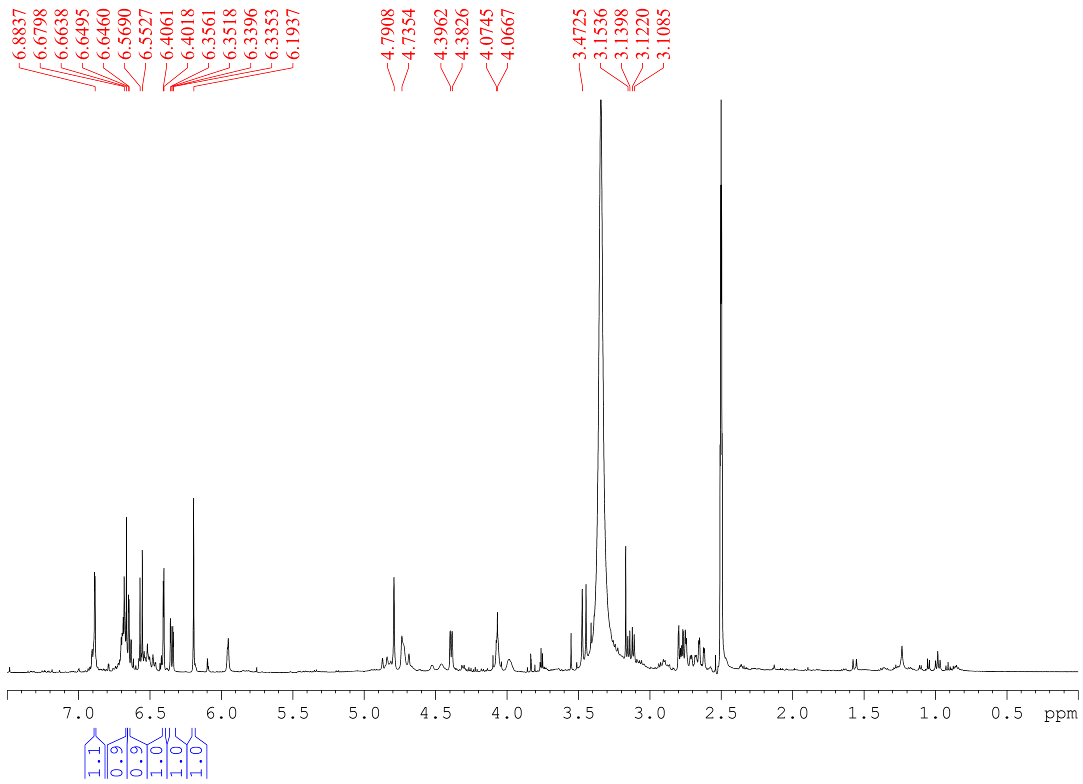

Figure S20. ^{1}H NMR spectrum (500 MHz, DMSO-d_6) of **5**

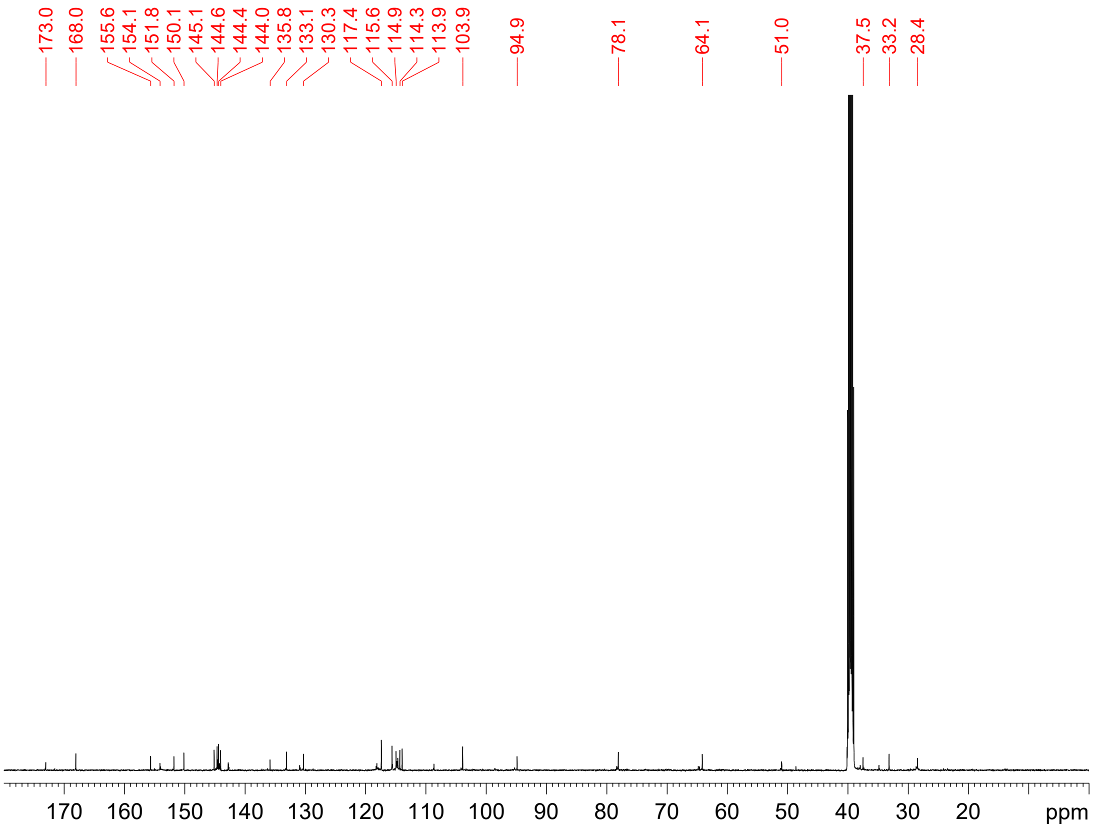

Figure S21. ^{13}C NMR spectrum (125 MHz, DMSO-d_6) of **5**

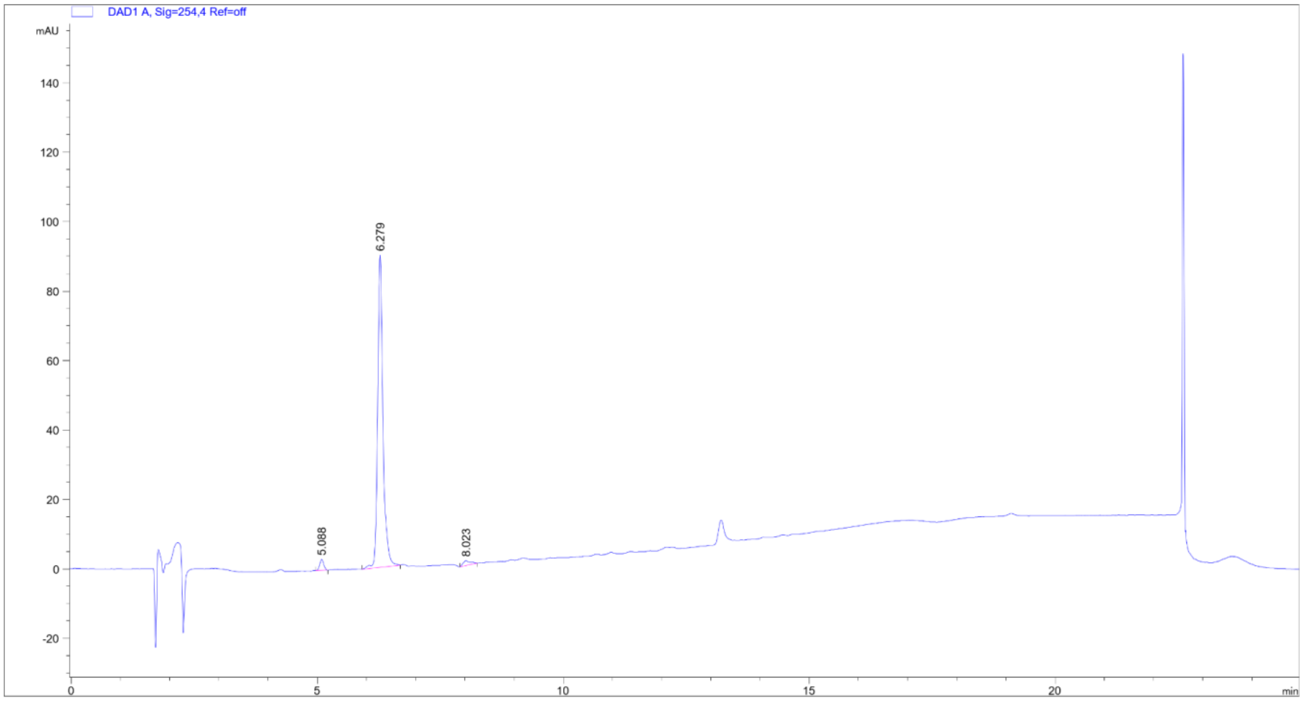

Figure S22. HPLC profile of **1**

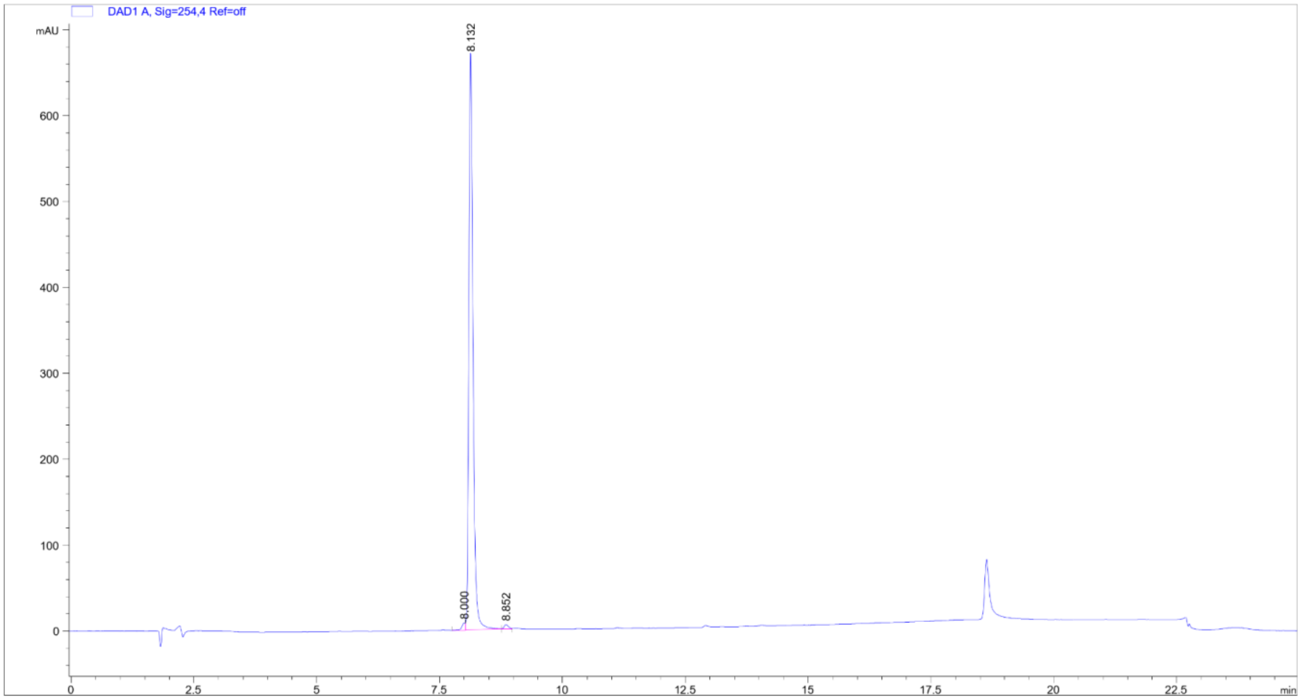

Figure S23. HPLC profile of **2**

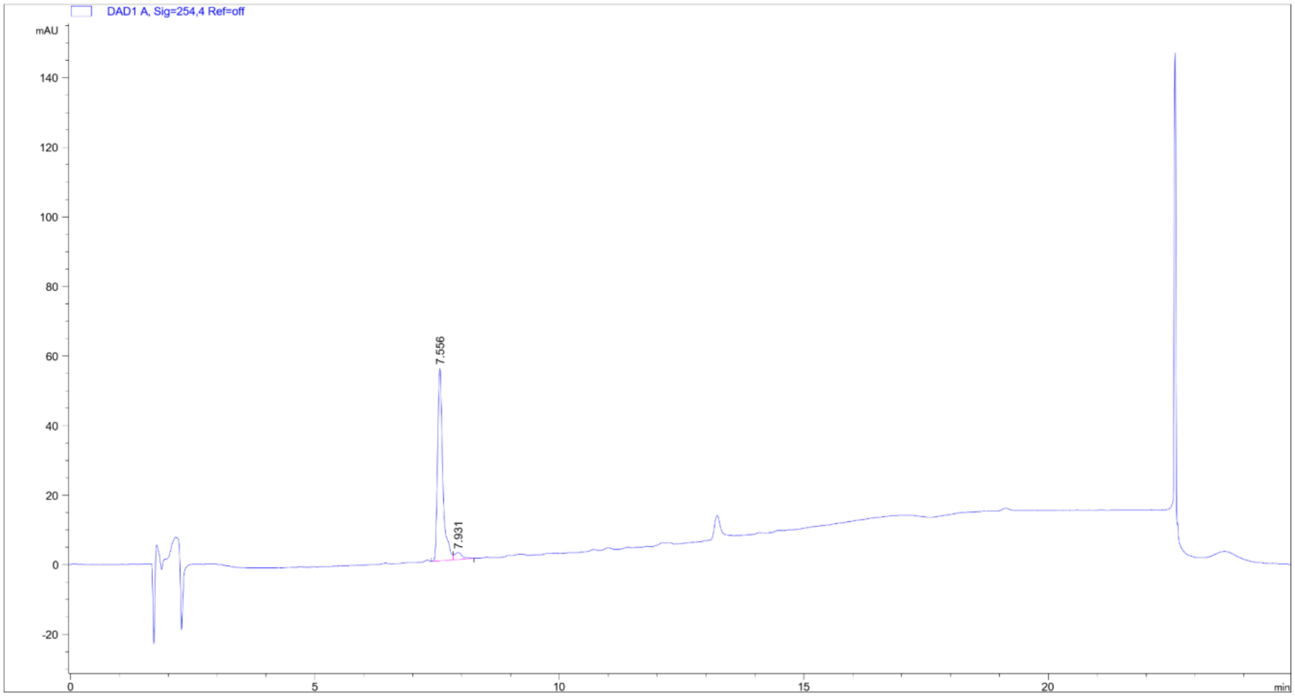

Figure S24. HPLC profile of **3**

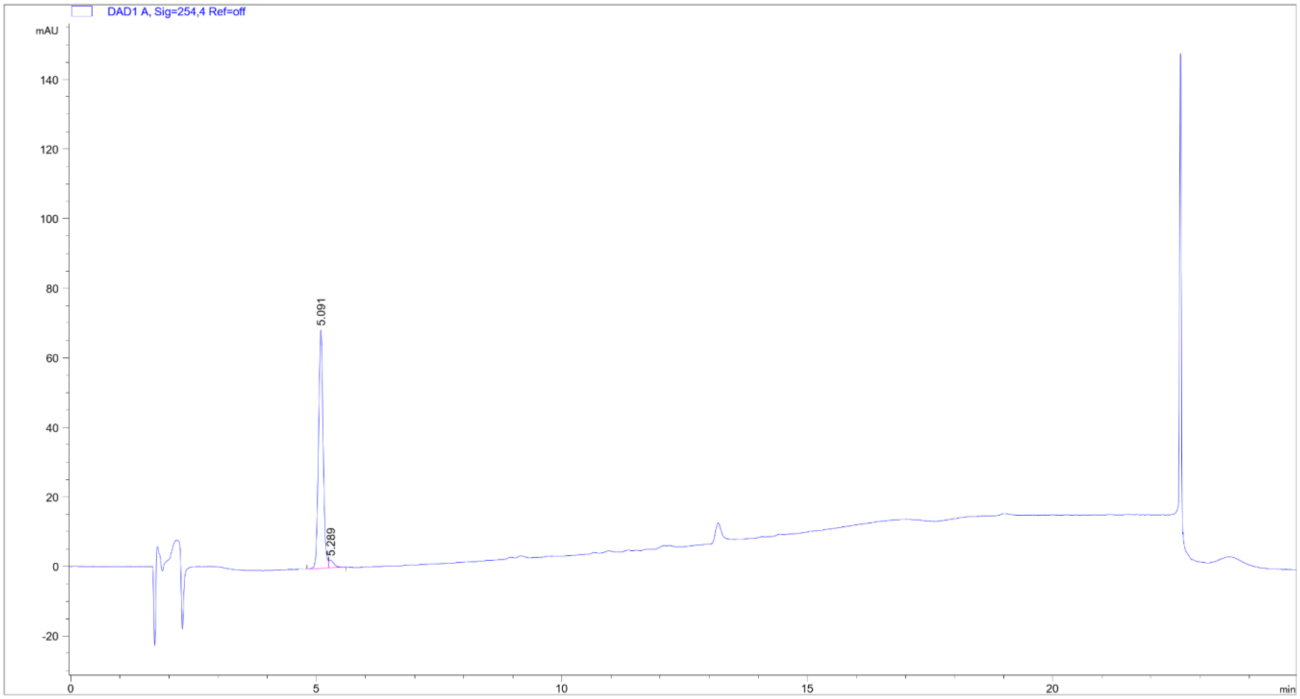

Figure S25. HPLC profile of **4**

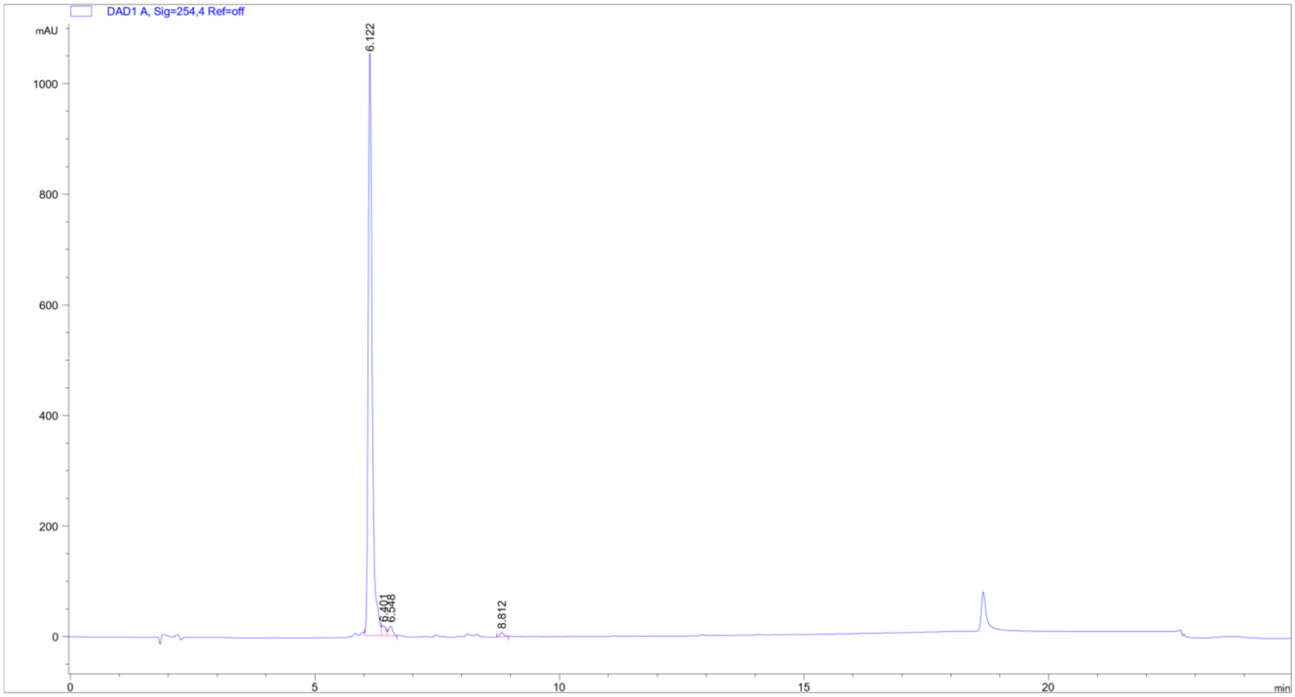

Figure S26. HPLC profile of **5**

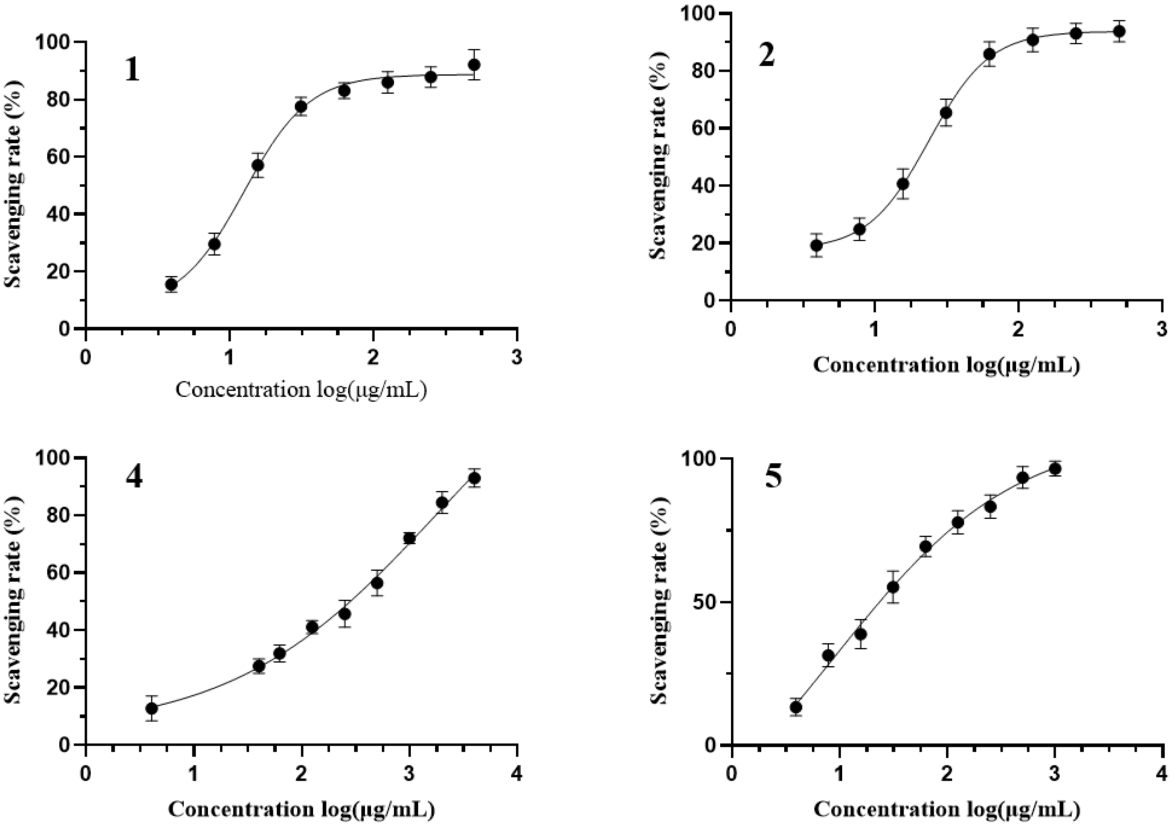

Figure S27. ABTS radical-scavenging concentration-response curves (scavenging rate vs. log concentration) for compounds 1, 2, 4, and 5.

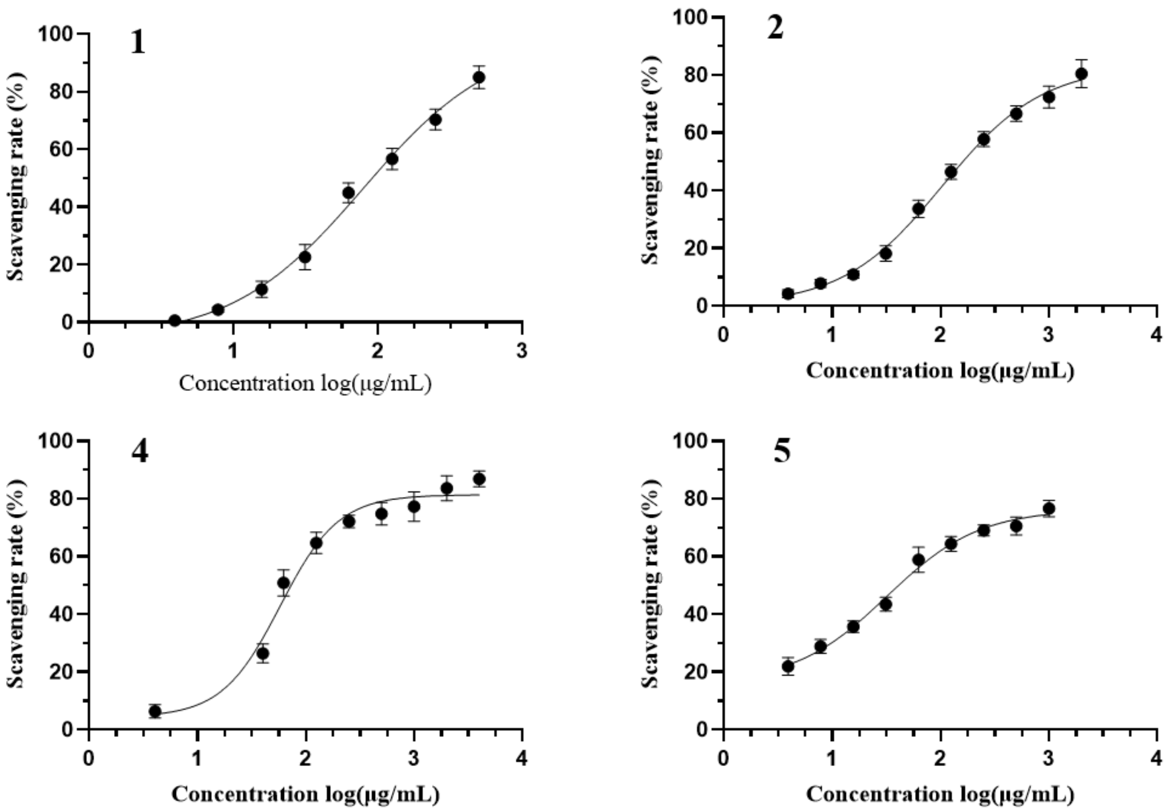

Figure S28. DPPH radical-scavenging concentration-response curves (scavenging rate vs. log concentration) for compounds 1, 2, 4, and 5.
